# Native Contact Density and Nonnative Hydrophobic Effects in the Folding of Bacterial Immunity Proteins

**DOI:** 10.1371/journal.pcbi.1004260

**Published:** 2015-05-27

**Authors:** Tao Chen, Hue Sun Chan

**Affiliations:** Departments of Biochemistry, of Molecular Genetics, and of Physics, University of Toronto, Toronto, Ontario M5S 1A8, Canada; UNC Charlotte, UNITED STATES

## Abstract

The bacterial colicin-immunity proteins Im7 and Im9 fold by different mechanisms. Experimentally, at pH 7.0 and 10°C, Im7 folds in a three-state manner via an intermediate but Im9 folding is two-state-like. Accordingly, Im7 exhibits a chevron rollover, whereas the chevron arm for Im9 folding is linear. Here we address the biophysical basis of their different behaviors by using native-centric models with and without additional transferrable, sequence-dependent energies. The Im7 chevron rollover is not captured by either a pure native-centric model or a model augmented by nonnative hydrophobic interactions with a uniform strength irrespective of residue type. By contrast, a more realistic nonnative interaction scheme that accounts for the difference in hydrophobicity among residues leads simultaneously to a chevron rollover for Im7 and an essentially linear folding chevron arm for Im9. Hydrophobic residues identified by published experiments to be involved in nonnative interactions during Im7 folding are found to participate in the strongest nonnative contacts in this model. Thus our observations support the experimental perspective that the Im7 folding intermediate is largely underpinned by nonnative interactions involving large hydrophobics. Our simulation suggests further that nonnative effects in Im7 are facilitated by a lower local native contact density relative to that of Im9. In a one-dimensional diffusion picture of Im7 folding with a coordinate- and stability-dependent diffusion coefficient, a significant chevron rollover is consistent with a diffusion coefficient that depends strongly on native stability at the conformational position of the folding intermediate.

## Author Summary

In order to fold correctly, a globular protein must avoid being trapped in wrong, i.e., nonnative conformations. Thus a biophysical account of how attractive nonnative interactions are bypassed by some amino acid sequences but not others is key to deciphering protein structure and function. We examine two closely related bacterial immunity proteins, Im7 and Im9, that are experimentally known to fold very differently: Whereas Im9 folds directly, Im7 folds through a mispacked conformational intermediate. A simple model we developed accounts for their intriguingly different folding kinetics in terms of a balance between the density of native-promoting contacts and the hydrophobicity of local amino acid sequences. This emergent principle is extensible to other biomolecular recognition processes.

## Introduction

The study of proteins that fold in an apparent two-state-like manner [1] has led to tremendous advances in protein folding biophysics [2, 3]. In line with the consistency [4] and minimal frustration [5] principles, the energy landscapes of these proteins may be pictured as smooth funnels with little ruggedness [6–8]. However, the consistency between local and nonlocal interactions is never perfect [4]. Frustration exists [5] in biomolecules and can sometimes serve important biological functions [9]. It is physically intuitive that energetically favorable nonnative interactions can occur [10]. Through improved experimental techniques, nonnative interactions are now known to be more prevalent than previously appreciated [11, 12]. From a fundamental biophysical standpoint, a better understanding of the presence and absence of nonnative interactions is key to deciphering biomolecular recognition and to assessing our grasp of basic protein energetics [13].

**Fig 1:**
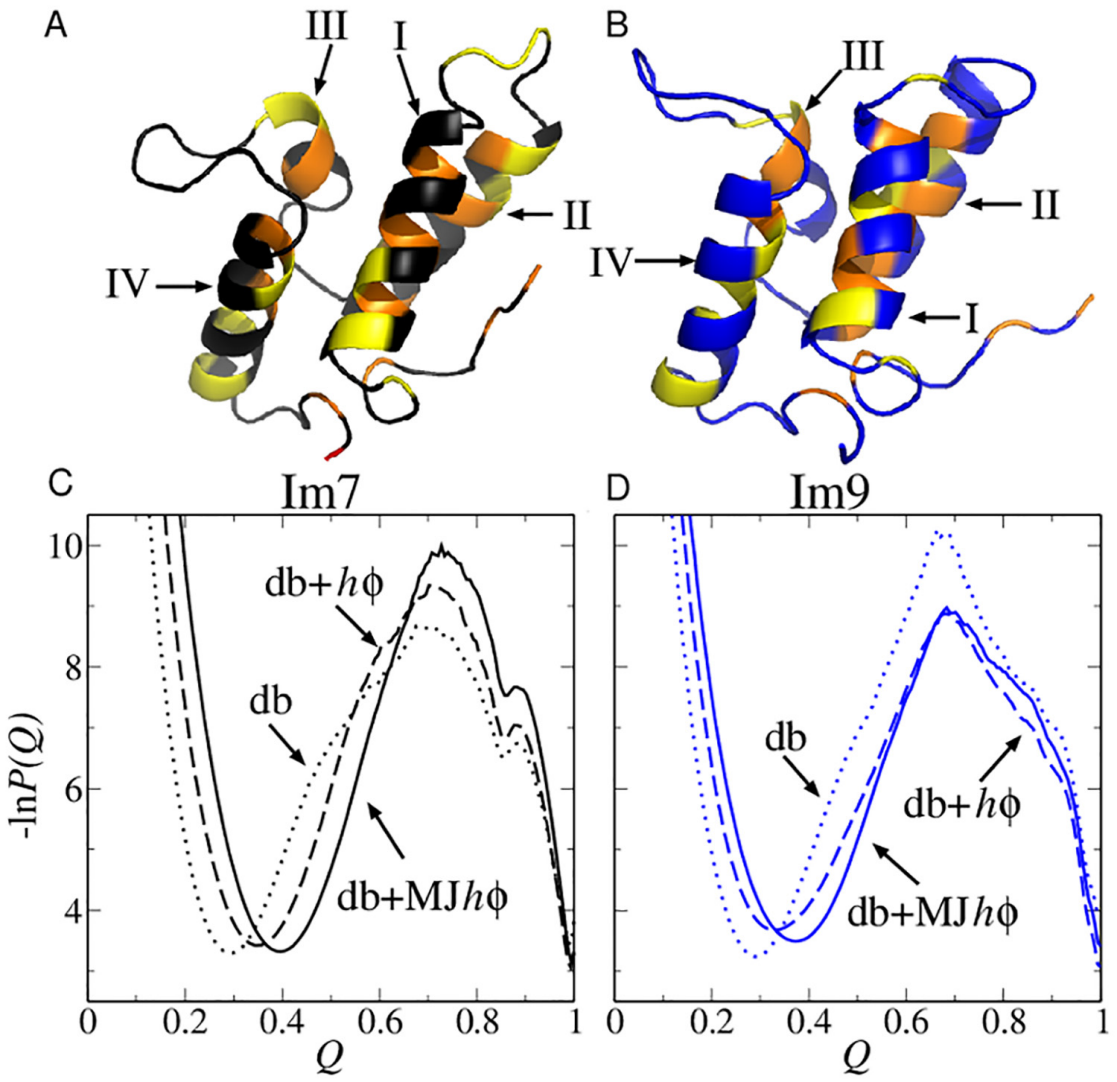
Structures and folding thermodynamics of Im7 and Im9. The ribbon diagrams (top) depict the PDB structures of (A) Im7 (PDB ID: 1AYI) and (B) Im9 (PDB ID: 1IMQ). The positions of four types of strongly hydrophobic residues (M, F, I, L) are shown in orange whereas those of four types of largely nonpolar residues but have weaker hydrophobicities (V, W, Y, A) are shown in yellow. Other residue positions are shown in black (for Im7) or blue (for Im9). Each structure contains four helices (I, II, III, and IV). The bottom panels show free energy profiles −ln *P*(*Q*) for Im7 (C) and Im9 (D) computed using three different models around each model’s transition midpoint.

As one of the earliest definitive examples of nonnative effects in single-domain proteins, the folding kinetics of bacterial immunity protein Im7 and its homolog Im9 are well characterized [14, 15]. Despite their very similar native structures (Fig 1A and 1B), a large body of experimental work demonstrates that Im7 folds via an intermediate stabilized by nonnative contacts, whereas Im9 folding is essentially two-state [16–22]. The relative simplicity of the Im7/Im9 system makes it well suited for an informative case study. Unlike some of the larger proteins (number of residues *n* ≳ 100) such as cytochrome c [23] and ribonuclease A [24] that fold in a more complex manner [25], Im7 and Im9 folding is not complicated by a heme or disulfide bonds. Indeed, in view of many single-domain proteins that can fold with no apparent nonnative effects, the nonnative interactions in Im7 are likely a consequence of functional constraints [26, 27]. It is noteworthy in this connection that the biological functions of Im7 and Im9 are evolutionarily related by promiscuous interactions [28] that are probably underpinned by nonnative excited-state conformations [29].

Theory and computation have provided valuable insights into the Im7/Im9 system. Experimental Φ-values were used as constraints in conformational sampling to derive putative folding transition states of these proteins [27, 30]. The results suggest a functional origin for the nonnative interactions in Im7 [27]. In a separate effort, an equilibrium intermediate state was predicted for Im7 using a Gō-like model that assumes no favorable nonnative interaction [31]. However, although topological frustration and heterogeneity in contact density can, in some cases, lead to kinetic and equilibrium folding intermediates in the absence of favorable nonnative interactions [32–34], a subsequent computational study indicates that Im7 folding cannot be explained by native-centric interactions alone [26]. Instead, nonnative effects arising from “localized frustration” [35] was seen as necessary for rationalizing the peculiar behaviors of Im7 [26]. Consistent with this finding as well as with experiment, a sequential stabilization algorithm for predicting folding pathway was not able to reach the Im7 native structure because of kinetic trapping; but the same algorithm was successful in accessing the Im9 native structure [36].

A clear kinetic difference between Im7 and Im9 is manifested by their chevron plots of logarithmic folding and unfolding rates versus denaturant concentration [11]. The folding arm of the Im7 chevron at pH 7.0 and 10°C exhibits a significant rollover, whereas that of the Im9 does not [16, 18–20]. The present study addresses this basic distinction between Im7 and Im9 by direct simulations of folding/unfolding rates. Because each chevron plot is a summary of kinetic and thermodynamic data from a large set of folding/unfolding trajectories [13], it is not yet practical to employ all-atom molecular dynamics [37, 38] for the extensive computation necessary to produce model chevron plots. Moreover, current molecular dynamics forcefields are probably insufficient to rationalize highly cooperative folding behaviors such as that of Im9 because the forcefields tend to over-predict nonnative effects [38, 39]. Therefore, as an interim method that has been applied elsewhere [40–42], we develop tractable explicit-chain coarse-grained models [43] to tackle the chevron behaviors of Im7 and Im9, as these behaviors have not been addressed by direct simulations to date. We model nonnative effects using “hybrid” formulations that augment structure-based native-centric interactions with physics-based, sequence-dependent transferrable energy terms [44, 45]. Limitations notwithstanding, this approach has been accounting for an increasing number of experiments [13, 43, 46–51]. By comparing nonnative interactions that do [52] and do not [47, 49] reflect the variation of hydrophobicity among nonpolar residues [53], we find that the difference between the Im7 and Im9 chevrons is well rationalized by nonative interactions involving large hydrophobic residues.

The present study addresses also the relationship between conformational diffusion and folding intermediates. Diffusion is a useful concept [54–59] in describing physical effects of solvent and internal friction in folding [60–63]. Whereas mild internal friction likely arises from the particulate nature of the solvent [62] and correlated dihedral rotations along the polypeptide [63], elevated internal friction in compact chains [60] can emerge more generally from topological frustration [32, 33] and favorable nonnative interactions [10, 54]. As discussed below, the Im7 chevron rollover in our model is associated with a coordinate- and stability-dependent coefficient of one-dimensional diffusion, with a strong anticorrelation between native stability and diffusion rate at the position of the transiently trapped intermediate. Notably, the smallest diffusion coefficients at these trapped positions can be orders of magnitude smaller than those encountered in two-state-like folding.

## Results/Discussion

We study three classes of coarse-grained chain models for Im7 and Im9. The rationale for the models—termed db (desolvation-barrier), db+*hϕ*, and db+MJ*hϕ*—are detailed in *Methods*. The db models are purely native-centric, whereas the other two are hybrid models [13] that allow for sequence-dependent nonnative hydrophobic interactions based on either homogeneous or heterogeneous nonnative energies. The nonnative interaction strength between any pair of hydrophobic residues is taken to be the same in the homogeneous db+*hϕ* models. We compare this simple approach [49]—which does not account for effects of mutations among hydrophobic residues—to the heterogeneous db+MJ*hϕ* models that utilize a Miyazawa-Jernigan (MJ) statistical potential [52] for the nonnative interactions among different hydrophobic pairs. To compare models on an equal footing, the *average* hydrophobic interaction strength in the heterogeneous db+MJ*hϕ* models is chosen to be identical to that of the homogeneous db+*hϕ* models.

### The difference between Im7 and Im9 folding is not apparent in the model proteins’ *Q*-dependent free energy profiles

**Fig 2:**
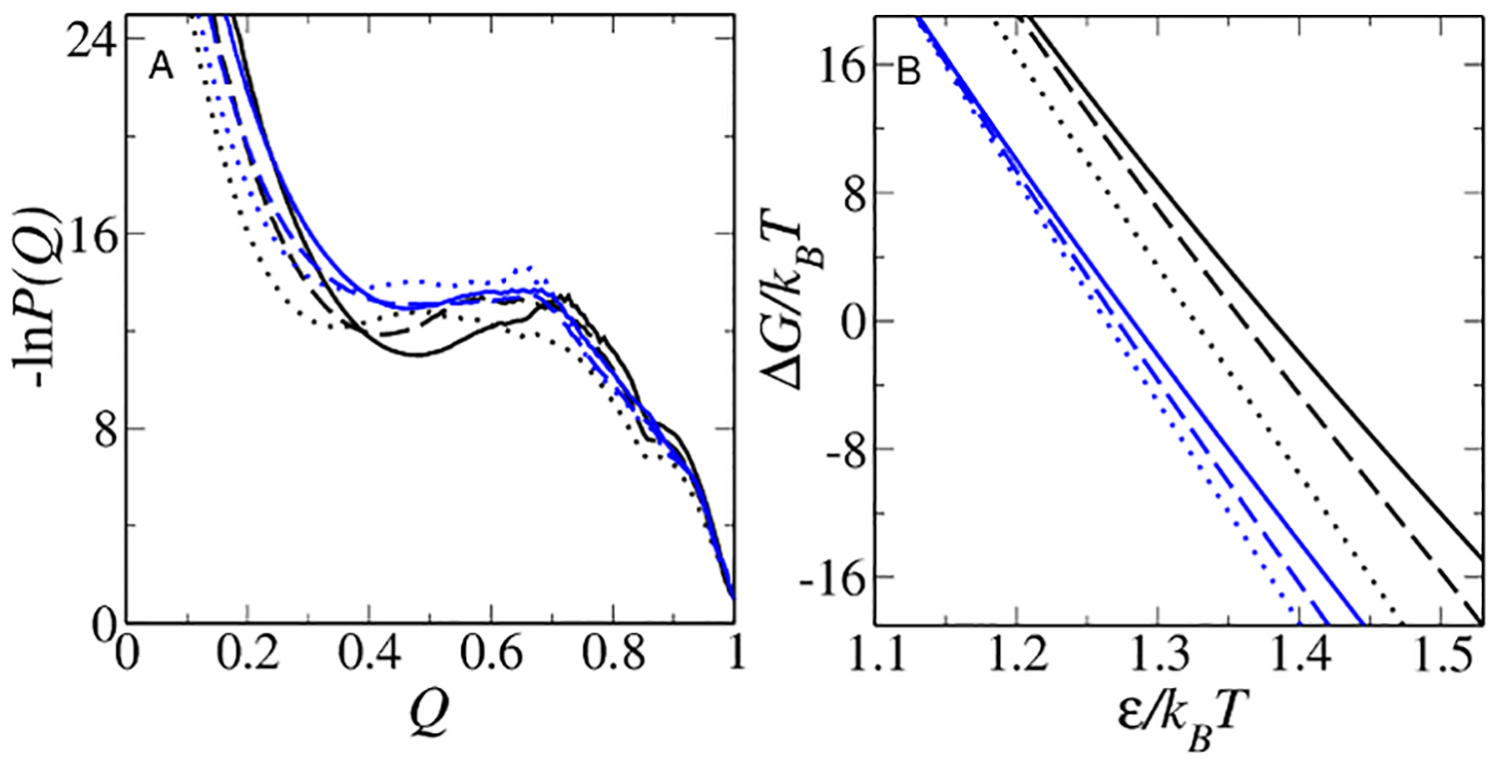
Modeling native stability changes. (A) Simulated Im7 (black) and Im9 (blue) free energy profiles at Δ*G*/*k*_*B*_ *T* values that equal to the experimental stabilities of the proteins at zero denaturant. (B) For all six models studied, Δ*G*/*k*_*B*_ *T* varies approximately linearly with inverse temperature 1/*T*. Results for db, db+*hϕ* and db+MJ*hϕ* in (A) and (B) are plotted using the line styles in Fig 1. The Δ*G*/*k*_*B*_ *T* values here and in subsequent figures are computed by identifying conformations with *Q* ≤ *Q*_D_ = 48/154, 57/154, and 61/154 as the Im7 unfolded states, respectively, in the db, db+*hϕ* and db+MJ*hϕ* models; and conformations with *Q* ≥ *Q*_N_ = 151/154 as the Im7 folded state in all three models. The corresponding criteria for the Im9 unfolded states are *Q*_D_ = 50/164, 56/164, and 61/164; and the Im9 folded state is defined by *Q*_N_ = 159/164 for all three models.

The equilibrium free energy profiles computed near the models’ transition midpoints (Fig 1C and 1D) show no dramatic difference between Im7 and Im9. The free energy barrier is lower for Im7 than for Im9 in the db models (dotted curves); but this trend is reversed when the nonnative interactions in the db+*hϕ* and db+MJ*hϕ* models are included (dashed and solid curves). Nonnative interactions in these models slow down folding for Im7 but speed up folding for Im9. Unlike previous Im7 models that exhibit a significantly populated equilibrium intermediate [26, 31] (which is apparently not quite in line with the success of two-state fitting of experimental equilibrium data for wildtype Im7 [22]), folding in our models is thermodynamically two-state as their folding/unfolding barriers under midpoint conditions are quite high (≳ 5*k*_*B*_ *T*, where *k*_*B*_ is Boltzmann constant and *T* is absolute temperature). The only hint of an Im7 folding intermediate is a small dip in the Im7 profiles (Fig 1C) at *Q* ≈ 0.85 that is absent in the Im9 profiles (Fig 1D). This feature by itself is no definitive evidence for complex folding kinetics, however. Under much stronger folding conditions, folding in our models becomes downhill [64]. Now even less difference is seen in Fig 2A between the equilibrium free energy profiles of Im7 and Im9 under zero-denaturant conditions (Δ*G*/*k*_*B*_ *T* ≈ −10.5 and −12.0, corresponding to the experimental folding free energy of approximately −24.9 kJ mol^−1^ for Im7 [19] and −28.2 kJ mol^−1^ for Im9 [15] at pH 7.0 and 10°C; see Fig 2B).

### The main difference between Im7 and Im9 chevron plots is rationalized by heterogeneous nonnative hydrophobic interactions

**Fig 3:**
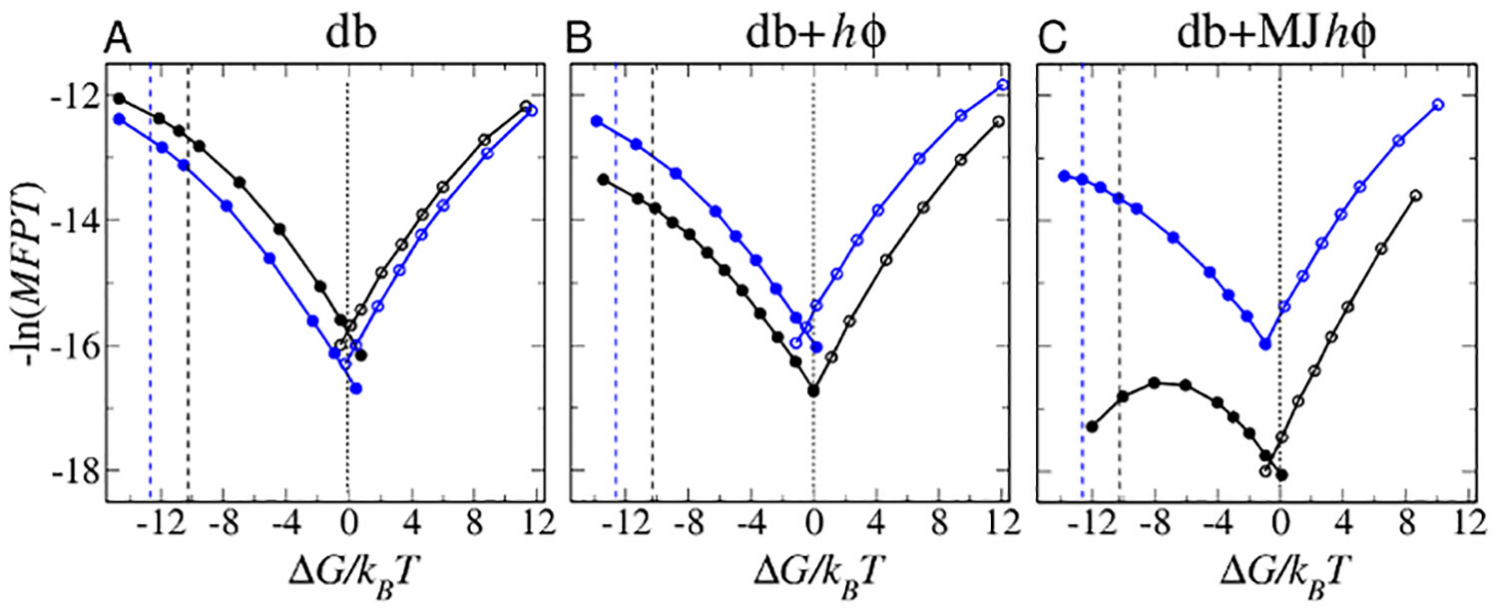
Model chevron plots. Negative logarithm of mean first passage time (*MFPT* in units of number of simulation time steps) of folding (filled circles) and unfolding (open circles) for Im7 (black data points) and Im9 (blue data points) as a function of Δ*G*/*k*_*B*_ *T* of the given model. Fitted curves are merely guides for the eye. The Δ*G*/*k*_*B*_ *T* values corresponding to the experimental stability at zero denaturant are marked by vertical dashed lines using the same color code, whereas the Δ*G*/*k*_*B*_ *T* = 0 transition midpoints are marked by black dotted lines. The kinetic criteria for folding and unfolding are identical to those in Fig 2 for equilibrium Δ*G*/*k*_*B*_ *T*. Each Im7 *MFPT* data point in the (A) db, (B) db+*hϕ* and (C) db+MJ*hϕ* models is an average over the folding or unfolding times of 3,200, 2,532–3,200, and 604–3,200 trajectories, respectively. The corresponding numbers of trajectories for Im9 *MFPT* data points are 3,046–3,200, 2,513–3,200, and 3,200.

**Fig 4:**
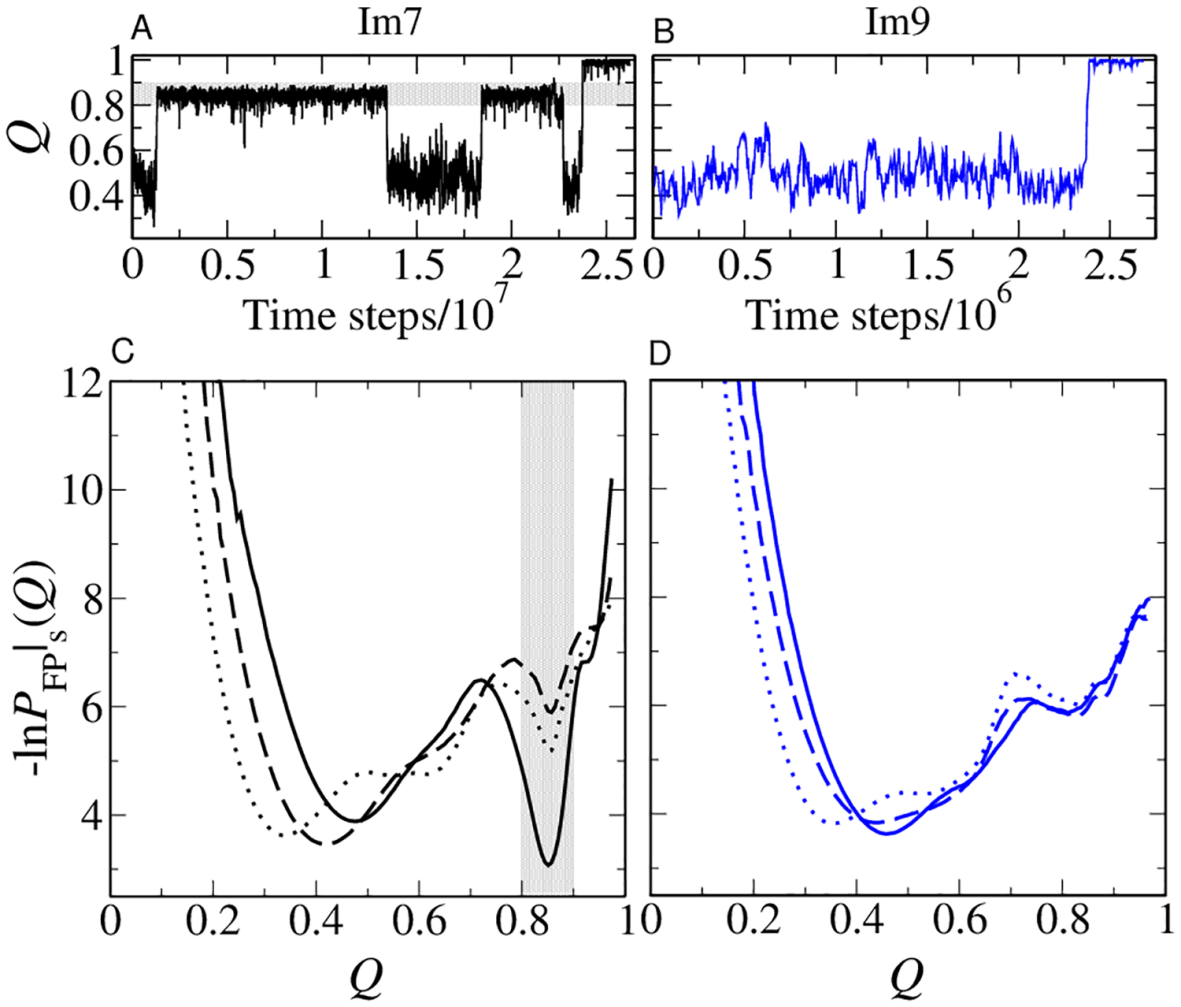
Transiently trapped conformations. (A, B) Examples of folding trajectories of Im7 (A) and Im9 (B) simulated using the db+MJ*hϕ* model under zero-denaturant conditions (*ϵ*/*k*_*B*_ *T* = 1.48 and 1.39 respectively). Transiently trapped conformations with *Q* ≈ 0.8–0.9 are observed for the Im7 trajectory [highlighted by horizontal gray shading in (A)] but not for the Im9 trajectory in (B). (C, D) Kinetic FP profiles [59] in the db, db+*hϕ*, and db+MJ*hϕ* models (same line styles as Fig 1) for Im7 (C) and Im9 (D). The approximate range of *Q* values for the conformations constituting the transiently trapped Im7 intermediate in the db+MJ*hϕ* model is indicated by the vertical gray band in (C). The number of trajectories used to computed the kinetic FP profiles in the three models are, respectively, 1,600, 1,240, and 1,139 for Im7 and 1,600, 1,600, and 3,200 for Im9.

The approximate linearity of native stability versus interaction strength *ϵ*/*T* (Fig 2B) allows Δ*G*/*k*_*B*_ *T* to be used as a proxy for denaturant concentration [42] in model chevron plots. Fig 3 shows that the folding-arm rollover and lack thereof, respectively, in the experimental chevrons for Im7 and Im9 at pH 7.0 and 10°C [16, 18–20] is captured by the db+MJ*hϕ* but not the db and db+*hϕ* models, suggesting that the Im7 rollover arises from the strong nonnative interactions among the large hydrophobic residues as modeled by db+MJ*hϕ* (S1 Fig). The difference between Im7 and Im9 folding cannot be explained by native interactions alone (as in db) or the more generic nonnative hydrophobic effects in db+*hϕ*. The chevron rollover in the db+MJ*hϕ* Im7 model is a consequence of transient yet long-lived trapped conformations at *Q* ≈ 0.85 (Fig 4A), which do not appear in Im9 folding under similarly strong folding conditions (Fig 4B).

An overview of Im7 and Im9 folding kinetics is afforded by their kinetic profiles, which show a deep minimum at *Q* ≈ 0.85 for Im7 (Fig 4C) but not for Im9 (Fig 4D). Determined from folding trajectories alone [59], kinetic profiles are more useful than free energy profiles for identifying folding intermediates. The Im7/Im9 difference is not apparent from the free energy profiles because, on one hand, kinetic trapping is minimal when folding is only weakly favored (Fig 1C). On the other hand, when folding is strongly favored (Fig 2A), the contribution from folding trajectories to an equilibrium profile is overwhelmed by that from unfolding trajectories, viz., the resident time in the folded state is much longer than that in the unfolded and intermediate states. Consequently, the deep well at *Q* ≈ 0.85 in Fig 4C translates to merely a small kink around the same *Q* value in Fig 2A.

A physical account of the main difference between Im7 and Im9 folding kinetics is thus provided. Many mutational experiments are rationalized below as well. Because of their simplicity, however, db+MJ*hϕ* models are limited in some respects. For instance, the midpoint folding rate of Im7 is ≈ 1/5 that of Im9 in this model (Fig 3C); but the experimental midpoint rate of Im7 (≈ 1.2–3.0 s^−1^ [19, 65]) is ≳ 40 times that of Im9 (≈ 0.03 s^−1^ [15, 20]). Moreover, whereas the folding and unfolding arms of the simulated chevron plots are quite symmetric around the transition midpoint, experimental unfolding rate exhibits a much weaker denaturant dependence than folding rate [16, 18–20]. These drawbacks are typical of topology-based models [42], which are more apt for folding than for unfolding kinetics [43, 66]. But this limitation has little bearing on our analysis of folding kinetics. Improved modeling likely requires special stability-enhancing energies that have minimal effects on folding kinetics [67, 68]; but such efforts are outside the scope of the present work.

### Contact pattern of the computed Im7 folding intermediate is consistent with experimental inference

**Fig 5:**
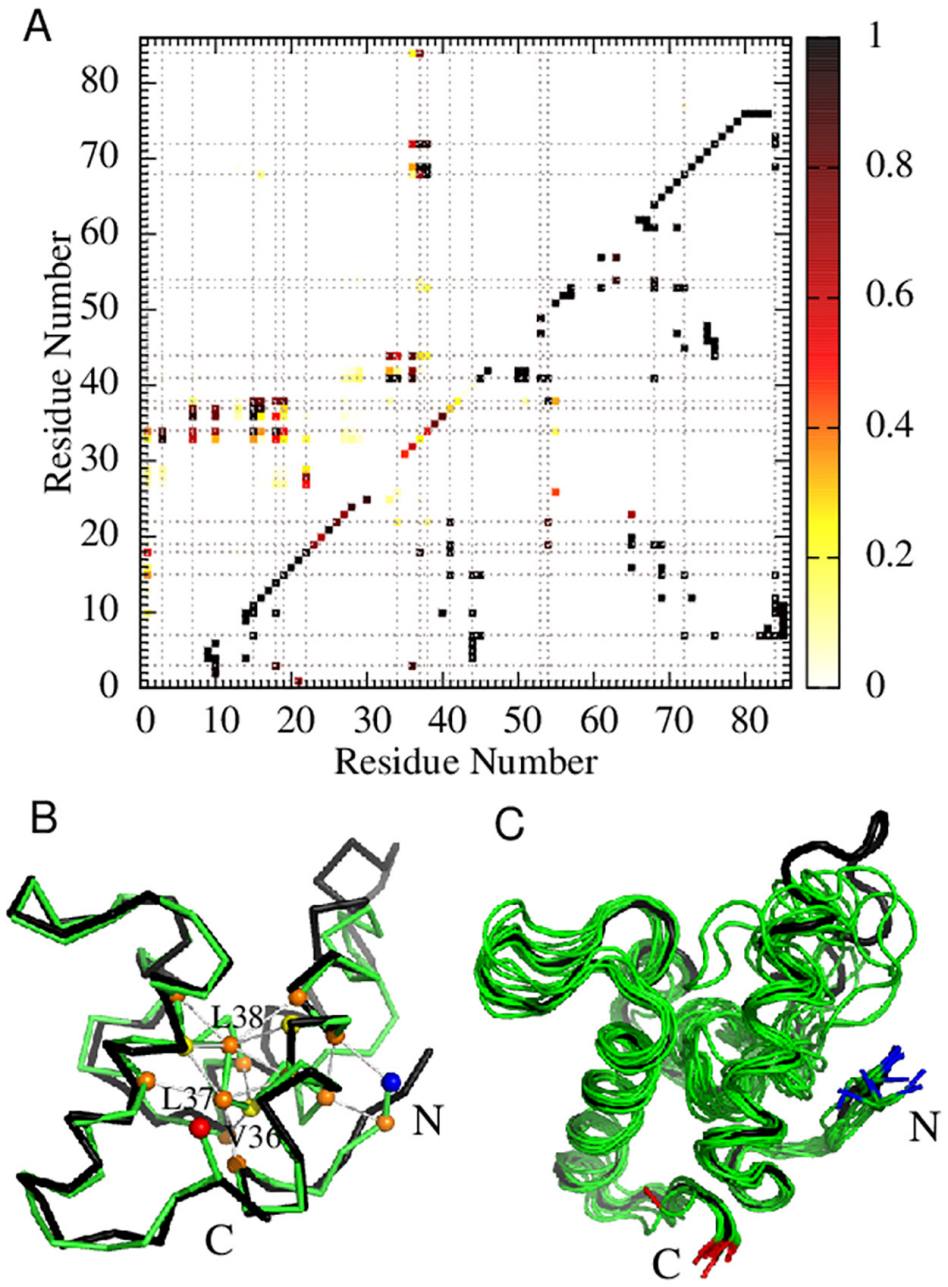
Structural properties of the simulated Im7 folding intermediate in the db+MJ*hϕ* model. (A) Native (lower right) and nonnative (upper left) contact probability maps (color scale on right) for Im7 conformations with 0.8 < *Q* < 0.9 along folding trajectories simulated under the strongly folding conditions in Fig 4. The maps provide occurrence probabilities of individual contacts in the putative intermediate-state ensemble that are normalized for the 0.8 < *Q* < 0.9 conformations along folding trajectories. The grey dotted lines mark the M, F, I, and L residues along the Im7 sequence. (B) One such Im7 conformation at *Q* = 0.844 (green *C*_*α*_ trace) is compared with the PDB structure (black trace). In the intermediate conformation (green trace), the N- and C-termini are marked, respectively, by the blue and red spheres. Hydrophobic residues that participate in significant nonnative interactions are marked as orange or yellow spheres (same color code as that in Fig 1A). A significant nonnative interaction is marked by a gray line between a pair of residues if the pair is not a native contact yet their spatial separation in the conformation shown is less than 8.0 Å and their interaction energy is stronger (more negative) than −1.0. The marked nonnative contacts are M1–L18, L3–L34, I7–L37, F15–L37, F15–L38, V16–L38, L18–L34, L19–L38, V36–F41, V36–I44, L37–V69, L37–I72, L38–I68, and L38–V69. (C) A collection of randomly chosen Im7 intermediate conformations (green traces). Included for reference is the PDB structure (black trace).

Structural properties of our simulated Im7 intermediate (Fig 5) are largely in agreement with mutagenesis experiments, which indicate that the intermediate is stabilized by nonnative interactions between Helix IV and the open end of the Helix I-Helix II hairpin involving residues L3, I7, F15, V16, L18, L19, L34, L37, L38, F41, V42, I68, and I72 [19]. Notably, 12 of these 13 residues are involved in the most populated 20 nonnative hydrophobic contacts (with > 80% probability of occurrence) in the Im7 intermediate simulated using db+MJ*hϕ* (Fig 5A, upper triangle). The only exception is V42, for which the most probable nonnative contact V36–V42 has nonetheless a 73% occurrence probability in the simulated intermediate. Among the 20 most probable nonnative contacts in the simulated Im7 intermediate, three are between the N-terminal segment and Helix II [L3–V33 (94%), L3–L34 (85%), I7–V36 (92%)], eight are between Helices I and II [F15–L34 (92%), F15–V36 (99%), F15–L37 (97%), F15–L38 (85%), V16–L37 (92%), V16–L38 (80%), L19–L38 (80%), L18–L34 (96%)], four are between different residues in Helix II [V33–F41 (98%), L34–F41 (99%), V36–F41 (90%), V36–I44 (82%)], and five are between Helices II and IV [L37–V69 (99%), L37–I72 (96%), L38–I68 (99%), L38–V69 (99%), L38–I72 (91%)]. Helix III hardly contributes to the intermediate-stabilizing nonnative contacts in the model. The most likely nonnative contact in the intermediate ensemble that involves Helix III, L38–L53, has an occurrence probability of only 17%.

Our computed probabilities of contacts are in line with experiments indicating that Helices I and IV are fully formed but Helix II is partly formed in the Im7 intermediate [14]. In Fig 5A, intrahelical contacts between residues *i*, *i* + 4 are present but less probable for Helix II (residues 32 to 45) than for Helices I and IV (residues 12 to 26 and 65 to 78). Experiment indicates also that Helix III is absent [14] but it is present in our simulated Im7 intermediate. This limitation of the model is likely related to its simple treatment of native interactions. Nonetheless, in agreement with experiment, amino acid substitutions in Helix III result only in small changes in folding rate in the db+MJ*hϕ* model (see below).

A snapshot of the simulated Im7 intermediate state is shown in Fig 5B wherein each of the highlighted nonnative contacts has ≥ 80% occurrence probability except M1–L18 (56%) in the Im7 intermediate ensemble (Fig 5C). All except one (V42) of the 13 residues identified by mutagenesis experiments (see above) to be stabilizing the Im7 intermediate are represented in the highlighted nonnative contacts. We have verified that structures very similar to the C_*α*_ intermediate conformation in Fig 5B are physically realizable by constructing a corresponding atomic structure [69] with added sidechains [70] (S2 Fig).

Our simulated Im7 kinetic intermediate is stabilized by nonnative interactions (S1 Fig). As such, it is diametrically different from the equilibrium intermediates simulated using purely native-centric models [31] with heterogeneous Gō energies [71]. Instead of being a product of nonnative effects, equilibrium intermediates in such Gō-like models arise from their reduced folding cooperativity [72], which can lead to three-state-like free energy profiles for Im7 and Im9 (S3 Fig); but such features are at odds with experiment.

### Kinetic effects of Im7 mutations

**Fig 6:**
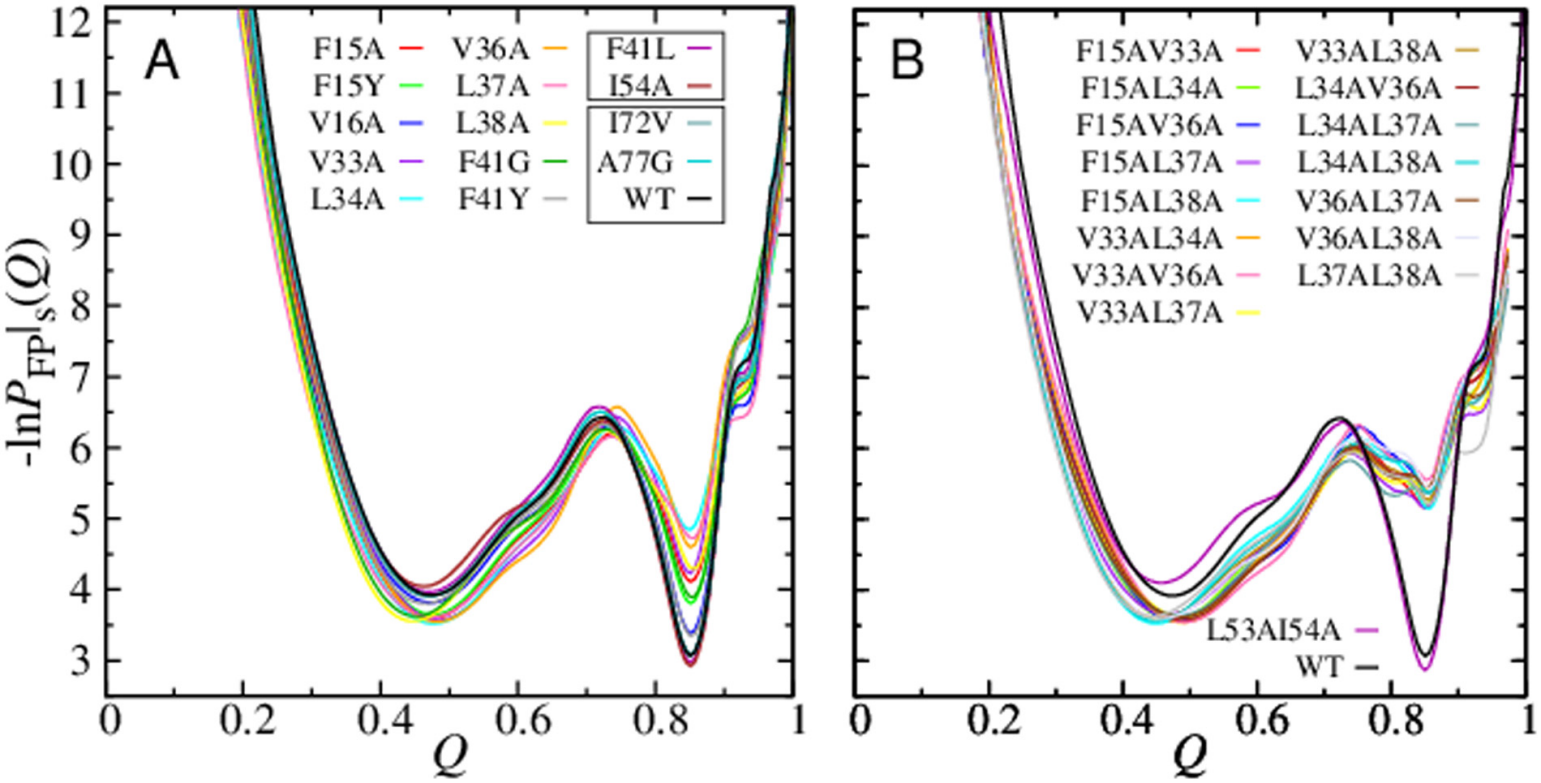
Kinetic effects of mutations in the db+MJ*hϕ* model for Im7. Kinetic FP profiles of single (A) and double (B) mutants under strongly folding conditions corresponding to zero denaturant in experiment. The reference WT profile is also shown. The depth of the kinetic trap at *Q* ≈ 0.85 for the single mutants in (A) increases in the following order: L34A (shallowest) < L37A < V36A < L38A < V33A < F15A < F41G < F15Y < V16A < F41Y < I72V < A77G < WT < F41L < I54A (deepest). The boxes in (A) enclose mutations that lead to very similar kinetic FP profiles. The kinetic FP profiles for the double mutant L53AI54A and the WT in (B) are also very similar.

Effects of select mutations in the db+MJ*hϕ* model for Im7 are examined through their folding kinetic profiles [59] (Fig 6). Some mutations reduce the depth of the kinetic trap at *Q* ≈ 0.85 relative to that of the wildtype (WT) while others lead only to negligible changes. We compute also the rates of reaching the intermediate position at *Q* ≈ 0.85 and the folded state at *Q* = 0.98 from initially unfolded conformations. The former rate (≈ 3.9 × 10^−7^ for WT, in units of reciprocal number of time steps) varies little, whereas the latter overall folding rate (= 5.0 × 10^−8^ for WT) is sensitive to mutation. The overall folding rate correlates, albeit imperfectly, with the depth of the *Q* ≈ 0.85 minimum.

The general trend of variation of the simulated folding rates is largely in line with that of the experimental intermediate-to-native folding rates *k*_in_ [19] or *k*_IN_ [65] for the single mutants (= 238 s^−1^ for WT) in Fig 6A. For both simulation and experiment, folding rate remains essentially unchanged for three mutants (simulated rate in units of 10^−8^, experimental rate in s^−1^ [19, 65]): I54A (4.4, 200), I72V (5.0, 250), A77G (4.9, 235) [(5.0, 238) for WT]; and is speeded up for four mutants: F15A (30.7, 550), L34A (40.2, 1850), L37A (49.6, 450), L38A (31.1, 1600). Folding rate remains essentially unchanged experimentally but is speeded up in simulation for five mutants; nevertheless the simulated increase is less than that for mutants that fold faster in experiments: F15Y (19.3, 220), V16A (10.7, 220), V33A (19.7, 238), V36A (22.3, 190), F41Y (9.3, 186).

However, the present model cannot account for the dramatic experimental increase in folding rate and the disappearance of folding-arm rollover for F41L (*k*_in_ = 5000 s^−1^ [19], ≈ 21 times of that of WT) because F and L have similar MJ energies [52]. For this mutant, the simulated rate 3.6 × 10^−8^ is smaller than that of WT. Even mutating F to a non-hydrophobic in the model cannot produce the experimental effect of F41L, viz., the simulated rate for the F41G mutant is 2.68 times that of WT but is far from sufficient. To account for the dramatic impact of F41L, future theoretical studies will need to pursue subtle effects beyond our simple treatment of hydrophobicity, perhaps by considering energetics specific to aromatic residues [13, 73].

Consistent with experiment [14], L53A/I54A has a negligible kinetic effect on Im7 in our model (Fig 6B), which is in line with the small experimental Φ-values of ≈ 0.03–0.16 and *k*_in_ = 200 s^−1^ for L53 and I54 in Helix III [19]. In contrast, many double mutants with hydrophobicity-reducing substitutions in Helices I and II can dramatically destabilize the folding intermediate and thus speed up Im7 folding (Fig 6B). These predictions should be testable by future experiments. However, because mutations in our models change only the nonnative but not the native interactions, as it stands our approach cannot address mutations such as L18A/L19A/L37A that prevent Im7 folding [22].

### Im7/Im9 difference in native contact density and hydrophobicity of Helix II

The three-state kinetics of Im7 is related to its hydrophobic composition. Im7 has 32 hydrophobic residues (17 with stronger and 15 with weaker hydrophobicities; Fig 1) whereas Im9 has 28 (15 and 13 in the two categories). In Helix II, Im7 has two more hydrophobics (V33, V42) and the stronger L38 instead of the weaker V37 in Im9. In Helix IV, Im7 has I72 instead of Im9’s V71. Notably, V33, L38, and V72 are involved in 10 of the 20 most probable nonnative contacts in the simulated Im7 intermediate listed above.

**Fig 7:**
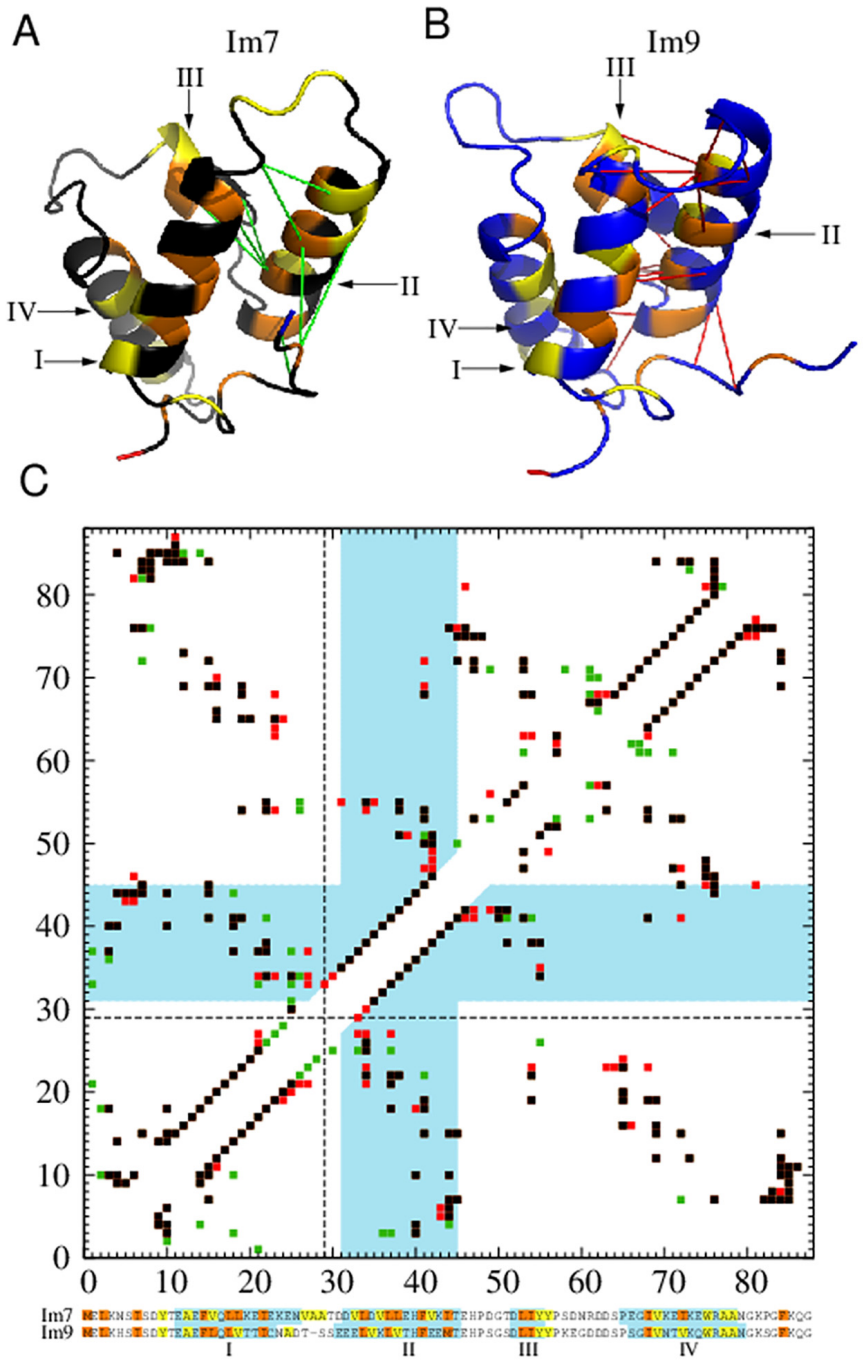
Local native contact density of Helix II is lower in Im7 than in Im9. (A) and (B) are the PDB structures shown with the color code in Fig 1 and depicted in a different orientation to highlight the native contacts of Helix II. The same color coding for hydrophobic residues is applied to the sequence alignment in (C) below. The eight green lines in (A) mark the native contacts involving Helix II that are found in Im7 but not in Im9, whereas the eighteen red lines in (B) mark the corresponding contacts that are in Im9 but not in Im7. Native contacts common to both proteins are not marked in (A) and (B). (C) Combined native contact maps for Im7 and Im9 using aligned residue numbering (bottom). The sequence alignment here follows that of Friel et al. [21], wherein Im9 residue number *i* is shifted to *i* + 1 for *i* > 27. The first of the shifted Im9 residues, at position 29, is marked by the dashed lines in the contact maps. Blue-shaded regions in the sequence alignment encompass residues belonging to the four helices as defined by the PDB. In the contact maps, native contacts common to Im7 and Im9 are plotted in black, whereas those belonging only to Im7 or only to Im9 are plotted, respectively, in green or red. Native contacts involving Helix II are those within the two L-shaped blue-shaded regions in the maps. The lower-right map follows the definition for native contacts in *Methods*; this map is the one used in the simulations as well as in the drawings (A) and (B). Included for comparison is the upper-left native contact map determined by the CSU software [74, 75].

Im7 and Im9 have nearly equal numbers of native contacts involving Helices I and IV (54 and 50, respectively, for Im7 and 53 and 49 for Im9). But the number of native contacts involving Helix II is 52 for Im7 (residues 32 to 45) and 62 for Im9 (residues 30 to 44). The native contact density of Helix II is thus appreciably lower for Im7 (52/14 = 3.71) than for Im9 (62/15 = 4.13). With lower local native-centricity and higher local hydrophobicity (Fig 7), Im7’s Helix II—which contains two more hydrophobic residues than Im9’s as shown by the sequences at the bottom of Fig 7 (see also discussion above)— is more prone to nonnative contacts than Im9’s Helix II. Indeed, Helix II is involved in all of the 20 most probable nonnative contacts in the simulated Im7 intermediate.

We emphasize that the critical factor here is the local native contact density of Helix II but not necessarily the overall native contact density of the protein. Im7 has fewer native contacts than Im9 (154 versus 164) in our models; yet the simulated Im7 intermediate remains essentially unchanged even if the number of Im7 native contacts is increased to 161 by using Swiss-PdbViewer [69] to construct additional contacts in its less ordered N-terminal region. Moreover, the trend seen here is not limited to our specific definition of native contacts. To assess the robustness of our inference, we have also applied the CSU software, which employs detailed analysis of interatomic contacts and interface complementarity to determine native contacts [74, 75]. Under the CSU criterion, the total number of native contacts is very similar for Im7 and Im9 (177 and 180 respectively; see upper-left map in Fig 7). Nonetheless, similar to the observation above, the local density of CSU-defined native contacts of Helix II is also appreciably lower for Im7 (59/14 = 4.21) than for Im9 (67/15 = 4.47).

Experiments on Im9 have shown that V37L/V71I and V37L/E41V/V71I can lead to three-state folding [15, 21] and folding-arm rollover at pH 7.0 and 10°C [21]. Computationally (S4 Fig), these mutations deepen somewhat the shallow minimum at *Q* ≈ 0.85 in the Im9 kinetic profile (A and C of S4 Fig). But the effect is insufficient to account for experimental data, indicating that further effort is needed to better model the balance between native and nonnative interactions in Im9. For instance, if the native interaction strength of L33 (which acts as a “gatekeeper” [76]) in Helix II was reduced, much deeper Im9 kinetic traps would develop for V37L/V71I and V37L/E41V/V71I (B and D of S4 Fig). Although our present model does not address mutational effects on native interactions, this result indicates nonetheless that L33 mutations that reduce the native interaction strengths (e.g., by substituting it with a less hydrophobic residue) may lead to less cooperative folding of Im9. This suggested behavior should be testable by future experiments.

The above analysis of the interplay between local native contact density and hydrophobicity suggests that the different folding kinetics of wildtype Im7 and Im9 may also be seen in variants of the homogeneous db+*hϕ* model (*K*_HP_ = 1 as defined in *Methods*) with stronger nonnative hydrophobic interaction strengths (*K*_HP_ > 1). Consistent with this idea, S5 Fig shows that a signficant folding intermediate population starts to develop at *K*_HP_ = 1.3 for Im7 but no corresponding folding intermediate is observed for Im9 at the same *K*_HP_. Two comments are in order here. On one hand, the result in S5 Fig from an alternate formulation of hydrophobicity reinforces our general notion that local native contact density and hydrophobicity are the main physical underpinnings for the Im7-Im9 kinetic difference. On the other hand, a strength of ≳ 1.3 for the homogeneous nonnative hydrophobic interaction is needed to achieve the desired Im7-Im9 difference, whereas the heterogeneous nonnative hydrophobic interaction strengths in the db+MJ*hϕ* model that produce a similar effect average only to 1.0 (see Methods; note that even at *K*_HP_ = 1.3, the minimum at *Q* ≈ 0.85 in (A of S5 Fig) is shallower than that in Fig 4C). Physically, *K*_HP_ ≳ 1.3 is problematic because it implies that nonnative interaction strength is ≳ 30% higher than native interaction strength. For this reason and considering the obvious limitation of the homogeneous approach that it cannot address effects of mutations among hydrophobic residues, the more refined db+MJ*hϕ* approach is adopted in the present study.

### Conformational diffusion in *Q* is extremely coordinate- and stability-dependent in the presence of a significant kinetic trap

The Im7/Im9 system is instructive in elucidating nonnative effects and kinetic trapping in the diffusion picture of protein folding [54–59]. Conformational diffusion models with a coordinate and stability-dependent diffusion coefficient on a one-dimensional free energy profile were constructed for two-state-like [57] and downhill [58] folding; but corresponding modeling for folding with a significant chevron rollover has not been much explored. In this regard, it is noteworthy that the rollover in our Im7 db+MJ*hϕ* model appears across only ≈ 8% variation in interaction strength (*ϵ*/*k*_*B*_ *T* = 1.37 and 1.48, respectively, for midpoint and zero denaturant). In contrast, rollover-like features for two-state-like and downhill folders emerge over much wider ranges of interaction strength [58].

**Fig 8:**
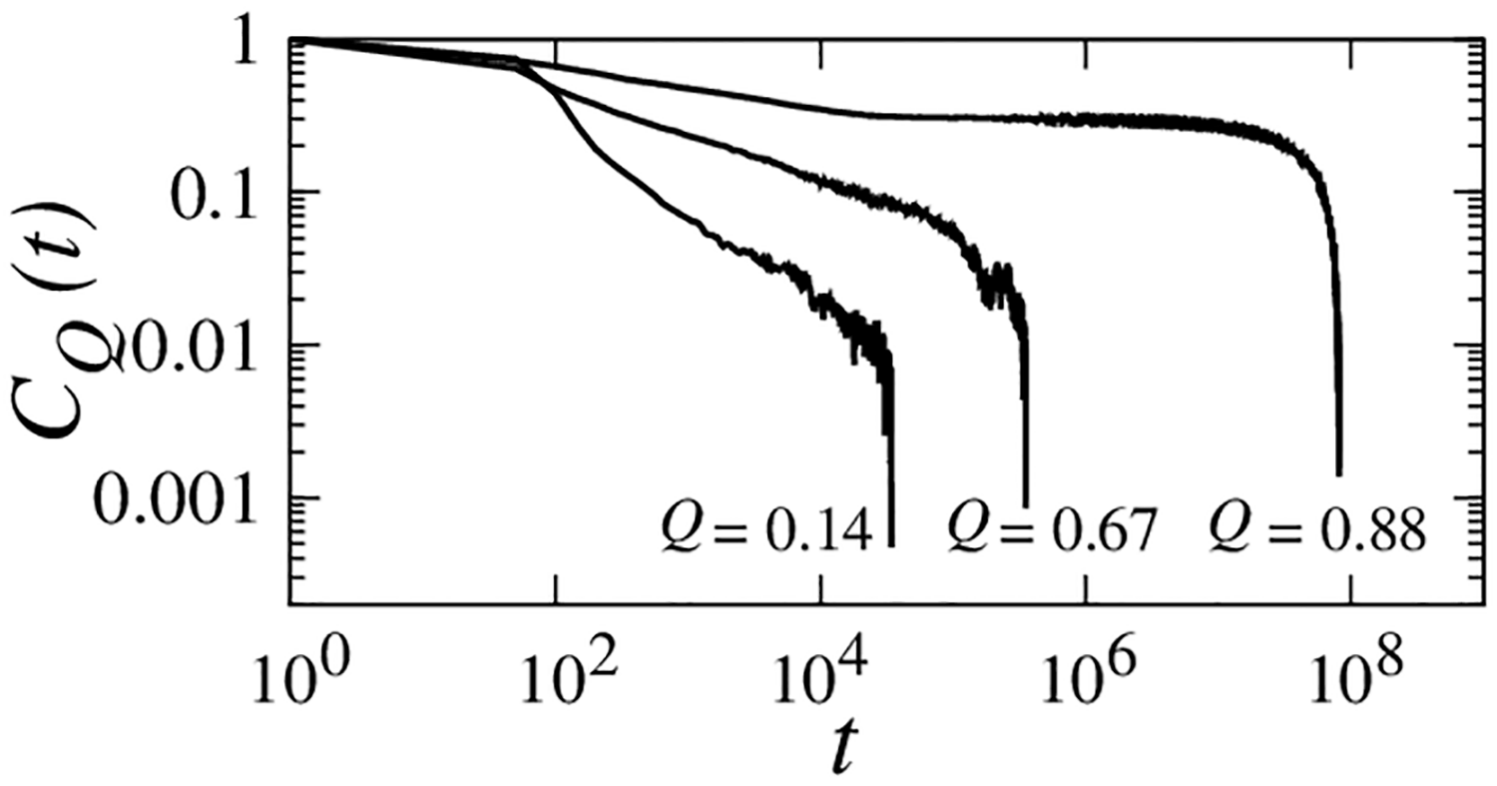
Autocorrelation functions of conformational diffusion *C*_*Q*_(*t*) for the db+MJ*hϕ* Im7 model computed near the experimental zero-denaturant stability Δ*G*/*k*_*B*_ *T* = −10.2 for three *Q* values. *C*_*Q*_(*t*)s were obtained by dynamic conformational sampling restrained to a small range of *Q* (see text). The *C*_*Q*_(*t*) functions for *Q* = 0.14 and *Q* = 0.67 were simulated using random initial conformations; the *C*_*Q*_(*t*) function for *Q* = 0.88 was obtained by initiating simulations from a conformation in the kinetically trapped intermediate state.

**Fig 9:**
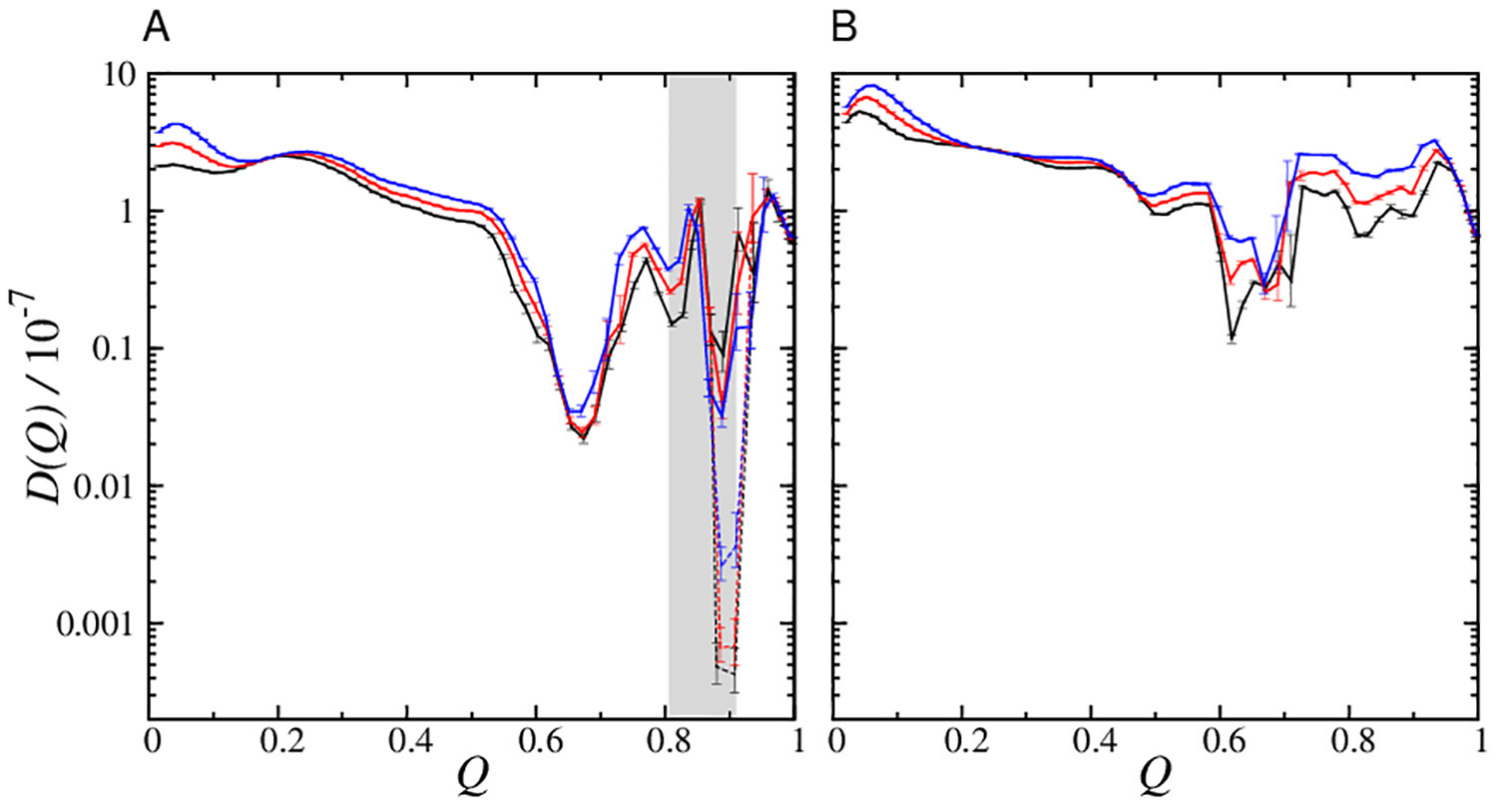
Coordinate- and stability-dependent conformational diffusion coefficients *D*(*Q*, Δ*G*) in the db+MJ*hϕ* model. *D*(*Q*) is computed for (A) Im7 at Δ*G*/*k*_*B*_ *T* = 2.1 (blue), −4.1 (red), −10.2 (black) as well as for (B) Im9 at Δ*G*/*k*_*B*_ *T* = 1.5 (blue), −5.9 (red), and −12.5 (black). For each Δ*G*/*k*_*B*_ *T* value, we applied 64 bias potentials centered at equally spaced *Q* values spanning *Q* ∈ [0, 1] to conduct restrained simulations of conformational dynamics to estimate *D*(*Q*), with 280 independent Langevin trajectories starting with random conformations simulated for each bias potential. Error bars were derived from standard deviation of the mean of autocorrelation times. Lines connecting data points are merely guides for the eye. The shaded area in (A) indicates the approximate *Q* values of the kinetically trapped Im7 intermediate (see Fig 4). Because simulated relaxation time in this region is highly sensitive to the starting conformation, to provide a lower-bound estimate of the diffusion coefficient, for each Δ*G*/*k*_*B*_ *T* we considered two alternate *D*(*Q*) values that were estimated from conformational dynamics initiated from kinetically trapped conformations instead of random conformations (eight long trajectories were simulated per *D*(*Q*) value). These estimated lower bounds on *D*(*Q*) are shown in (A) as the lower data points at two *Q* positions (*Q* ≈ 0.85–0.90). To guide the eye, these additional data points are connected to the rest the *D*(*Q*) function by dotted lines.

The restraining-potential method [56, 58] in *Methods* is used to compute *Q*- and Δ*G*-dependent autocorrelation function *C*_*Q*_(*t*) (Fig 8) and diffusion coefficient *D*(*Q*) (Fig 9). The restraining-potential method directly addresses the escape probability from a given *Q*. Rather than seeking a good fit by Bayesian analysis [55], we adopt this method to explore possible limits of the diffusion picture by testing the consistency between diffusive accounts of restrained and unrestrained chain kinetics.

The most notable Im7/Im9 difference presents itself around the Im7 kinetic trap at *Q* ≈ 0.8–0.9. Here a dramatic deepening of a dip in *D*(*Q*) with increasing native stability is seen for Im7 but not for Im9, whereas *D*(*Q*) for other *Q*-values is not very sensitive to Δ*G* (Fig 9). Achieving numerical convergence of the computed *D*(*Q*) in the *Q* ≈ 0.85 region of Im7 is difficult because of kinetic trapping. To delimit theoretical possibilities, we obtain lower and upper bounds of *D*(*Q*) for Im7 in this region, respectively, by initializing restrained runs from kinetically trapped and random conformations (Fig 9).

**Fig 10:**
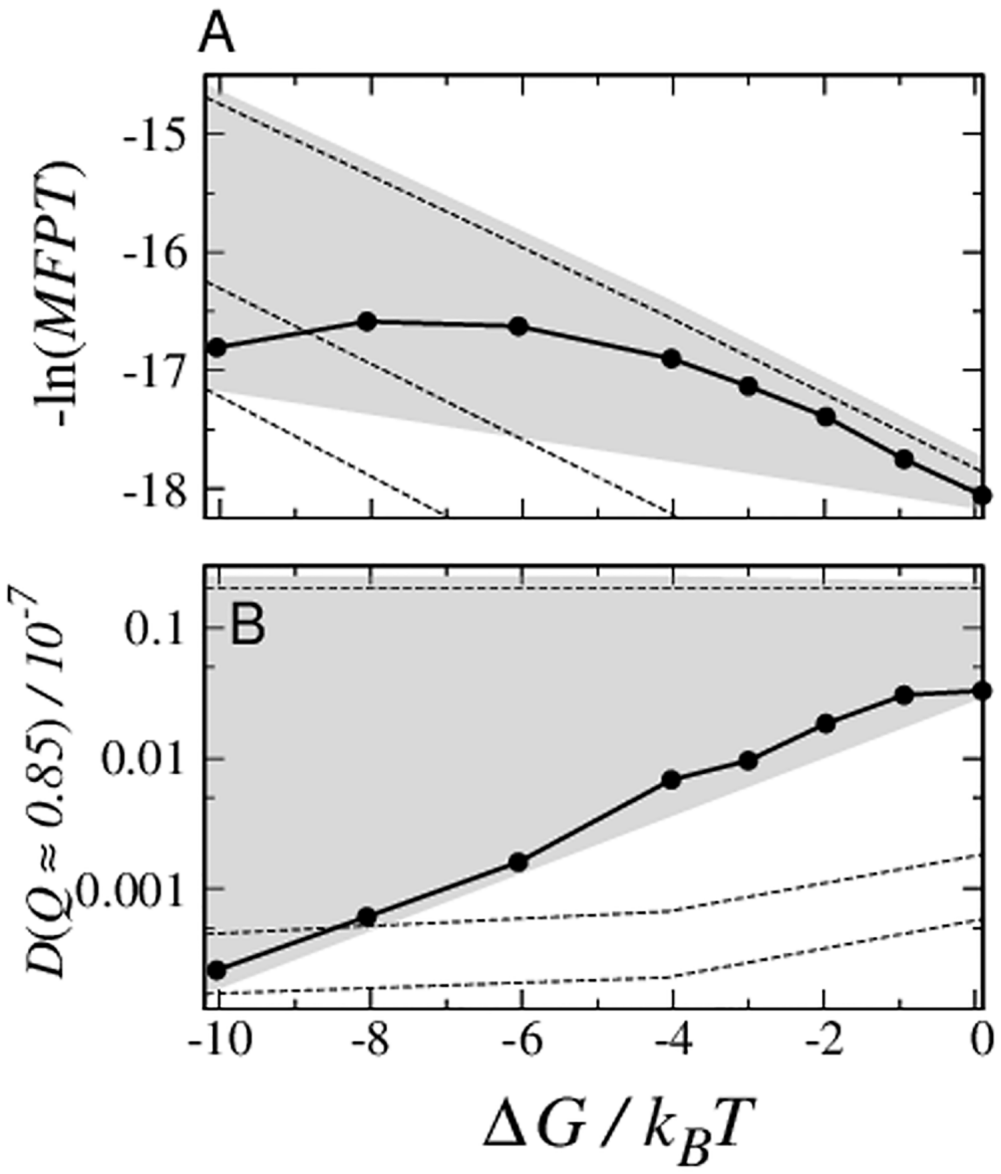
Chevron rollover in the diffusion picture of Im7 folding. (A) The folding arm of the Im7 chevron plot in Fig 3C is shown here again by the filled circles connected by solid lines. The dashed lines show the negative logarithm of stability-dependent folding *MFPT* computed analytically using the *D*(*Q*, Δ*G*) values in Fig 9A. The top (i), middle (ii), and bottom (iii) dashed lines are obtained, respectively, by (i) considering only the *D*(*Q*, Δ*G*) values estimated by simulations initiated from random conformation, i.e., not using the alternate lower-bound *D*(*Q*, Δ*G*) values for *Q* ≈ 0.85–0.90 at all, (ii) using the average lower-bound *D*(*Q*, Δ*G*) values for 0.81 < *Q* < 0.91, and (iii) using the minimum lower-bound *D*(*Q*, Δ*G*) values for the same range of *Q*. To faciliate comparison, all −ln(*MFPT*) values from the diffusion model are shifted by an overall additive constant of *c* = 2.7 so that the top dashed line may be compared with the explicit-chain chevron. This amounts to an overall re-scaling of the time units in the diffusion model. The shaded region shows the extent of possible folding-arm chevron behaviors. The upper boundary of this region was computed using the highest *D*(*Q*) values delimited by the error bars in Fig 9A. The lower boundary is constructed by joining the −ln(*MFPT*) values of the bottom dashed line [case (iii) above] at Δ*G* = −10.2*k*_*B*_ *T* with that at Δ*G* = 0 computed by using the minimum values delimited by the error bars for the *D*(*Q*) values estimated using random initial conformations. (B) Possible variation of the diffusion coefficient in the *Q* ≈ 0.85 region. The top, middle, bottom dashed lines and the shaded region in (B) show the *D*(*Q* ≈ 0.85, Δ*G*) values used to obtain the chevron behavior shown, respectively, by the top, middle, bottom dashed lines and the shaded region in (A). The filled circles connected by solid lines show the *D*(*Q* ≈ 0.85, Δ*G*) values needed to reproduce the trend of chevron rollover in explicit-chain simulations.

Im7 chevrons may now be computed in the diffusion model; but considerable variation ensues (shaded area in Fig 10A) because of numerical uncertainties. The rollover trend of the simulated Im7 chevron is among the predicted possibilities. However, when matched against explicit-chain kinetics, *D*(*Q*) is found to be underestimated by an overall factor of *e* ^2.7^ ≈ 15 (Fig 10), indicating that the method for computing *D*(*Q*) [56, 58] needs to be improved or that a one-dimensional diffusion perspective is of limited applicability here.

Despite these uncertainties, it is clear that a *D*(*Q*, Δ*G*) that decreases exponentially with Δ*G* at the trap position *Q* ≈ 0.85 (Fig 10B) is necessary to reproduce the folding-arm rollover for Im7 (Fig 10A, circles). The required variation of *D*(*Q*, Δ*G*) at this position, which spans two orders of magnitude, is reassuringly consistent with the lower bound estimated by initiating restrained runs from the kinetic trap. In the absence of such a strong dependence of *D*(*Q*, Δ*G*) on Δ*G*, the predicted folding arm would become essentially linear (top dashed line in Fig 10A). In the same vein and consistent with our explicit-chain results (Fig 3), no folding-arm rollover is produced by the diffusion model for Im9.

### Concluding remarks

To recapitulate, our explicit-chain models account physically for the strikingly different folding kinetics of Im7 and Im9 in terms of prevalent nonnative interactions among large hydrophobic residues in Im7 but not in Im9. The proteins’ different experimental chevron behaviors are rationalized by our simulation. The same phenomenon may also be described by a one-dimensional diffusion process with a very small and strongly stability-dependent diffusion coefficient at the position of the Im7 kinetic trapped intermediate.

Our model interaction schemes are tentative [13, 46]. For instance, possible contributions to nonnative interactions from electrostatic [48, 51, 77] and aromatic [73] effects are not taken into account. Nonetheless, by comparing different modeling schemes as controls and contrasting Im7 and Im9 behaviors under the same scheme, we arrive at a physical picture that is largely in agreement with experiment. As observed experimentally [65, 78], Helices I and IV are essentially formed while Helix II is partially formed in our simulated Im7 intermediate. Kinetic effects of many mutations in our model are consistent with experiment, including those involving Helix III (Figs 3–7), demonstrating the versatility of the hydrid modeling approach to nonnative effects [13].

Several limitations of our model are noted. In particular, the short Helix III is present in our simulated Im7 intermediate but experimentally that is apparently not the case [65, 78]. To address this issue, more sophisticated treatments of local conformational propensity [36, 79] and sidechain effects [13, 42] are probably needed. Indeed, the rich repertoire of experiments on the Im7/Im9 system, such as those on pH [18, 21] and temperature [15] effects, offers ample data for testing extensions of our models.

Perhaps the most useful insight from the present effort is that the peculiar folding kinetics of Im7 vis-à-vis that of Im9 is closely related to their difference in the balance between local native contact density and hydrophobicity. This principle embodies a competition between native topology and nonnative interactions [49] and is likely applicable to protein dynamics and biomolecular processes in general. As such, it should be examined in detail and extended to other forms of nonnative interactions in future investigations.

## Methods

### Explicit-chain models

Three related C_*α*_ chain models for Im7 and Im9 are considered, namely the db, db+*hϕ*, and db+MJ*hϕ* models. Common to these models is a set of native-centric interactions with desolvation barriers for each protein. Folding and unfolding kinetics is simulated by Langevin dynamics [80]. Desolvation barrier (db) is a robust feature in hydrophobic interactions [81] that tends to enhance folding cooperativity [40, 82]. Indeed, for some proteins such as ribosomal protein S6, C_*α*_ models with db lead to highly cooperative folding behaviors that are consistent with experiments [49] whereas models without db exhibit only weak folding cooperativity [76]. Here, following Ref. [80], the pairwise db energy is defined by a contact minimum well depth of *ϵ* = 1.0, a db height of *ϵ*_db_ = 0.1*ϵ*, and a solvent-separated minimum well depth of *ϵ*_ssm_ = 0.2*ϵ*.

The db model is purely native-centric with the total interaction potential, denoted here as *E*_N_, equal to *V*_total_ in Ref. [80]. The same interaction strength is applied to all native-centric interactions. The native contact sets for Im7 and Im9 are constructed using the same criterion [80]. A pair of residues *i*, *j* belongs to the native contact set if at least one pair of their non-hydrogen atoms, one from each residue, are less than 4.5 Å apart in the Protein Data Bank (PDB) structure. The PDB C_*α*_ separation between *i*, *j* is denoted by $r_{ij}^{\text{n}}$. The total number of native contacts in the set, ${\tilde{Q}}_{\text{n}}$, is equal to 154 and 164, respectively, for Im7 and Im9 (Fig 7). We have explored using alternate “flavored” native-centric interaction strengths [72, 83] in accordance with the residue-dependent contact energies in Ref. [71] but, interestingly, the resultant models for Im7 and Im9 fail to fold cooperatively.

### Homogeneous and heterogeneous nonnative interactions

Favorable nonnative interactions are included in db+*hϕ* and db+MJ*hϕ*. Using a hybrid formulation [13, 43–51, 84–92], the total interaction potentials *E*_T_ of these models are given by *E*_T_ = *E*_N_ + *E*_HP_, where $E_{\text{HP}}=\sum_{i}^{n}\sum_{j=i+4}^{n}K_{\text{HP}}\kappa_{ij}\exp[-{\left(r_{ij}-\sigma_{h\phi }\right)}^{2}]$ is the sum of sequence-dependent nonnative contact energies over *i*, *j* that are both hydrophobic (*hϕ*), defined to be the eight amino acids A, V, L, I, M, W, F, and Y [47]. *r*_*ij*_ is the C_*α*_ distance between *i*, *j* during simulation (1 ≤ *i*, *j* ≤ *n*, where the total number of residues *n* = 87 for Im7 and *n* = 86 for Im9); and *σ*_*hϕ*_ = 5.0 Å. The nonnative *hϕ* interactions in the db+*hϕ* model are homogeneous with *κ*_*ij*_ = −1.0 irrespective of hydrophobic residue type and *K*_HP_ = 1.0 as in Refs. [47, 49], whereas the nonnative *hϕ* interactions in the db+MJ*hϕ* model are heterogeneous, with *κ*_*ij*_ = Δ*ϵ*_*ij*_ where Δ*ϵ*_*ij*_ is the contact energy in Table V of Miyazawa and Jernigan [52] and *K*_HP_ = 1.8 such that the average *hϕ* energy *K*_HP_⟨*κ*_*ij*_⟩ over all possible 8 × 7/2 + 8 = 36 *hϕ* pairs is equal to −1.0 (the *K*_HP_ *κ*_*ij*_ values range from −0.216 for A-A to −1.584 for F-F). This average *hϕ* interaction energy of −1.0 is essentially maintained by the average *hϕ* energies over all possible nonnative *hϕ* contact pairs (defined below) for the Im7 and Im9 sequences in the db+MJ*hϕ* models. Those average energies are equal to −0.994 for wildtype Im7 (412 possible nonnative *hϕ* pairs) and −0.998 for wildtype Im9 (306 possible nonnative *hϕ* pairs).

MJ-type potentials [52, 71] are derived from the statistics of native contacts in the protein structure database. Because protein native structures do not contain many significantly unfavorable contacts, MJ potentials are not expected to describe repulsive interactions between amino acid residues with accuracy [93]. Nonetheless, they do provide a crude account of the relative strengths of favorable physical interactions between residues. In fact, it has long been known that MJ potentials for nonpolar pairs reflect the combined hydrophobicities of the two contacting residues [94, 95], as is illustrated by the good correlation (Fig 3b of [96]) between a set of MJ energies [71] and the experimental octanol-water transfer free energies of amino acids [53]. In this regard, although there are considerable variations among experimental hydrophobicity scales for all twenty types of amino acids [96, 97], a higher degree of consistency among different experimental scales is seen for the hydrophobic (nonpolar and non-charged) amino acids themselves [98]. Taking these considerations together, we view MJ energies between nonpolar residues as a reasonable coarse-grained model of the underlying physics of hydrophobicity. Thus, they should be applicable to favorable nonnative hydrophobic interactions and represent a more refined model than those with homogeneous hydrophobic interaction strengths.

In our models, two hydrophobic residues *i*, *j* that are not in contact in the native PDB structure are considered to be in a nonnative contact if |*i* − *j*| > 3 and *r*_*ij*_ < 8.0 Å (Fig 5). The total number of nonnative contacts in a conformation is denoted by *n*_HP_ (S1 Fig). All non-bonded energies in our models are temperature independent and pairwise additive. For simplicity, temperature dependence and nonadditity of interactions [99–102] are not considered here.

### Free energy profiles, kinetic profiles, and chevron plots

We consider a residue pair *i*, *j* in the native contact set to be in contact during the folding/unfolding simulation when $r_{ij}\leq r_{ij}^{\text{n}}+1.5$ Å; i.e., when *r*_*ij*_ is not larger than that of the db and therefore within the attractive well of the contact minimum. We use *Q*, the number of native contacts divided by ${\tilde{Q}}_{\text{n}}$, as progress variable of folding [103, 104]. A free energy profile in units of *k*_*B*_ *T* corresponds to −ln *P*(*Q*) where *P*(*Q*) is the normalized conformational population distribution as a function of *Q* (Figs 1 and 2). As was introduced before [59], the kinetic folding path (FP) profiles, −ln *P*_FP_|_s_(*Q*), is the negative logarithm of average fractional resident time *P*_FP_ as a function of *Q* along folding trajectories wherein the notation “|_s_” indicates that equal weight is assigned to every folding trajectory [59] (Figs 4 and 6). Chevron plots are simulated using change in native stability by varying the simulation temperature as a proxy for variation of denaturant concentration [105] (Fig 3). With a low Langevin viscosity, this approach is computationally efficient and is appropriate for our present purpose because the trend (shape) of model chevron rollover is apparently unaffected by variation over a wide range of Langevin viscosities [101]. Recent tests also indicate that the model chevron plots thus obtained are very similar to those simulated using more sophisticated coarse-grained sidechain models that account for denaturant dependence by experimental transfer free energies [13, 41, 42].

### Nonexplicit-chain models of one-dimensional conformational diffusion

We use the restraining (bias) potential method [55, 56, 58, 106] to estimate *Q*-dependent diffusion coefficients at different simulation temperatures (hence different free energies of folding Δ*G*). Following Ref. [55], a *Q*-dependent diffusion coefficient is given by

$$\begin{array}{c} D\left(Q\right)=\frac{\text{var}(Q)}{\tau_{\text{corr}}\left(Q\right)} \end{array}$$ (1)

for a given Δ*G*. Here the variance $\text{var}(Q)\equiv {\left\langle Q{\left(t_{0}\right)}^{2}\right\rangle }_{t_{0}}-{\left\langle Q(t_{0})\right\rangle }_{t_{0}}^{2}$, where ⟨…⟩_*t*_0__ denotes time averaging over different *t*_0_ values; the correlation time $\tau_{\text{corr}}(Q)=\int_{0}^{\infty }C_{Q}(t)dt$ where the autocorrelation function [54, 107]

$$\begin{array}{c} C_{Q}\left(t\right)=\frac{{\left\langle Q\left(t+t_{0}\right)Q\left(t_{0}\right)\right\rangle }_{t_{0}}-{\left\langle Q\left(t_{0}\right)\right\rangle }_{t_{0}}^{2}}{\text{var}(Q)} \end{array}$$ (2)

is *Q*-dependent. The var(*Q*) and *C*_*Q*_(*t*) for determining *D*(*Q*, Δ*G*) (Figs 8 and 9) are estimated using bias potentials $V_{\text{bias}}(Q,Q_{0})=K_{Q}{\tilde{Q}}_{\text{n}}^{2}{\left(Q-Q_{0}\right)}^{2}$, where the prescription in Ref. [108] is used to treat *Q* as a continuum variable. Unless specified otherwise, *K*_*Q*_ = 0.1*ϵ* is used with 64 different *Q*_0_ values for Im7 or Im9. This choice of *K*_*Q*_ is similar to that in Ref. [56] and serves to localize conformational fluctuations to Gaussian-like distributions (S6 Fig). *D*(*Q*) is quite insensitive to lowering *K*_*Q*_ by at least a factor of two (S7 Fig).

This method for determining *D*(*Q*) is exact if the diffusion process is truly governed by the Smoluchowski equation and *K*_*Q*_ is sufficiently large so that variation of free energy *G*(*Q*) within a constrained conformational ensemble is essentially linear in *Q*. The applicability of this approach to protein folding, however, hinges on whether the dynamics along *Q* is Markovian to a good approximation [55]. For protein folding, *D*(*Q*) estimated by the restraining-potential method does exhibit a weak dependence on *K*_*Q*_ [58]. We have checked our restraining-potential methodology against that of Xu et al. [58] by using a *K*_*Q*_ value that produces conformational distributions similar to theirs. Our *D*(*Q*) for chymotrypsin inhibitor 2 at transition midpoint matches well with theirs (S8 Fig). *D*(*Q*) can also be estimated using Bayesian analysis [55]. For one dipeptide system, the Bayesian-estimated *D*(*Q*) was verified to be very similar to that from restraining potentials [55]. Here we use only the restraining-potential method.

Once *D*(*Q*) is in place for a given native stability (free energy of folding) Δ*G*, the folding *MFPT* in our nonexplicit-chain models of one-dimensional conformational diffusion (Fig 10) is computed using the discretized form [59]

$$\begin{array}{c} {\left(MFPT\right)}_{D}=\sum_{Q=Q_{D}}^{Q_{N}}\;P_{\text{eq}}{\left(Q\right)}^{-1}\sum_{Q\prime =0}^{Q}\;P_{\text{eq}}\left(Q\prime \right)/D\left(Q\right) \end{array}$$ (3)

of the general formula [54, 109]

$$\begin{array}{c} {\left(MFPT\right)}_{D}=\int_{Q_{D}}^{Q_{N}}dQ\int_{0}^{Q}dQ\prime \;\frac{1}{D(Q)}\;\exp[\frac{G\left(Q\right)-G\left(Q\prime \right)}{k_{B}T}]\;, \end{array}$$ (4)

where *P*_eq_(*Q*) is the normalized equilibrium conformational population at *Q*. The boundary values *Q*_N_ and *Q*_D_ for the native (folded) and denatured (unfolded) states are the same as that in our explicit-chain simulations (Fig 2). Alternatively, *MFPT* can be computed using Kawasaki Monte Carlo (MC) dynamics by generalizing the formulation in Ref. [59] to coordinate-dependent *D*(*Q*), viz., the transition probability from *Q* to *Q* + *δQ* is now given by $A^{-1}\sqrt{D(Q)D(Q+\delta Q)}\exp[-\delta G(Q)/k_{B}T]$ where *δG* ≡ *G*(*Q* + *δQ*) − *G*(*Q*) and *A* is a constant. The above geometric mean $\sqrt{D(Q)D(Q+\delta Q)}$ may also be replaced by the arithmetic mean [*D*(*Q*) + *D*(*Q* + *δQ*)]/2; the two means are equal in the limit of *D*(*Q* + *δQ*) − *D*(*Q*) → 0. In addition to *MFPT*, Kawasaki MC is useful also for providing distribution of folding times and other properties of individual trajectories.

## Supporting Information

S1 Fig Nonnative hydrophobic interactions with physics-based heterogeneous strengths are needed to rationalize the Im7 folding intermediate. Results here are derived from kinetic folding trajectories simulated at Δ*G*/*k*_*B*_ *T* values corresponding to the zero-denaturant stabilities of the proteins being modeled. (A–C) Natural logarithm of contact probability (ln *P*_*ij*_, which is normalized for all conformations along folding trajectories, note that this normalization is different from that in Fig 5A). Native and nonnative contacts are shown, respectively, in the lower-right and upper-left (below and above the main diagonal). (A, B) Contact probability maps of Im7 conformations with 0.8 < *Q* < 0.9 simulated using the db+MJ*hϕ* (A) and db+*hϕ* (B) models. (C) Contact probability map of Im9 conformations with 0.6 < *Q* < 0.8 in the db+MJ*hϕ* model. It is clear from these maps that among conformations with *Q* ≈ 0.8, there are more nonnative contacts in the db+MJ*hϕ* model for Im7 than either the db+*hϕ* model for Im7 or the db+MJ*hϕ* model for Im9. (D) Number of nonnative hydrophobic contacts (solid curves, left vertical scale) and total nonnative hydrophobic interaction energy *E*_*HP*_ (dotted curve, right vertical scale) in the db+MJ*hϕ* model for Im7 (black curves) and Im9 (blue curves) as functions of *Q*.
(PDF)

S2 Fig Putative structural details of the simulated Im7 folding intermediate ensemble. (A) The red ribbon was generated from a typical C_*α*_ conformation in the folding intermediate of the db+MJ*hϕ* model (selected from Fig 5C of the main text) by first installing a complete backbone and sidechains (green ribbon) using the MaxSprout software (www.ebi.ac.uk/Tools/structure/maxsprout/) and then optimizing the resulting atomic conformation using the Swiss-PdbViewer software (http://spdbv.vital-it.ch/). Included for comparison is the native PDB structure of Im7 (1AYI, gray ribbon), wherein the four native helices are labeled as in Fig 1 in the main text. (B) The intermediate conformation with an optimized sidechain configuration. The red ribbon here is identical to that in (A). (C) Another conformation in the same folding intermediate ensemble. This conformation, which has a partially yet more substantially formed Helix II, is shown in the same format as that in (A). (D) The conformation in (C) with an optimized sidechain configuration, shown in the same format as that in (B).
(EPS)

S3 Fig Pure native-centric models with no favorable nonnative effects cannot rationalize the major difference in folding behavior between Im7 and Im9. (A, B) Free energy profiles of db models with homogeneous Gō potentials [black curve in (A) for Im7, blue curve in (B) for Im9] are compared with those with heterogeneous MJ Gō (native-centric) potentials [red curves in both (A) and (B)]. The profile for each model is computed near the model’s transition midpoint [*k*_*B*_ *T* = 0.76 for the black profile in (A), *k*_*B*_ *T* = 0.80 for the other three profiles in (A) and (B)]. The profiles show that the folding thermodynamics of models with homogeneous Gō interactions are two-state-like with a single barrier, whereas that of models with MJ Gō interactions are three-state-like with two barriers and an intermediate [free-energy minimum at *Q* ≈ 0.8 indicated by the rightmost vertical dotted line in (A) and (B)]. (C, D) Energy [*E*(*Q*), solid curves] and entropy [*S*(*Q*), dashed curves] in units of *k*_*B*_ *T* and *k*_*B*_, respectively, are shown as functions of *Q* for the models with homogeneous Gō and MJ Gō interactions [same color code as that in (A) and (B)]. The overlaying gray straight lines in (C) and (D), which are given by *y* = −240.0*Q* + 180.9 for (C) and *y* = −239.6*Q* + 180.2 for (D) where *y* is the vertical variable, are reference *Q*-dependences introduced for the analysis in (E) and (F). (E, F) Deviations of energy and entropy values from the reference *Q*-dependences (gray straight lines) in (C) and (D). Changes in energy and entropy [Δ*E*(*Q*) (solid curves) and Δ*S*(*Q*) (dashed curves)] relative to the common reference for Im7 (C, E) or the common reference for Im9 (D, F) for the models with homogeneous (black or blue curves) and MJ (red curves) Gō interactions are plotted using the same line styles as those in (C) and (D). [Note that the reference *Q*-dependences themselves now become the *y* = 0 horizontal gray lines in (E) and (F).] Position of peaks and minima along the free energy profiles in (A) and (B) are marked by the dotted vertical lines in (E) and (F) as well. By construction, the free energy profile of every model in (A) and (B) is given by *E*(*Q*)/*k*_*B*_ *T* − *S*(*Q*)/*k*_*B*_ of the model in (C) or (D) or, equivalently, Δ*E*(*Q*)/*k*_*B*_ *T* − Δ*S*(*Q*)/*k*_*B*_ of the model in (E) and (F). The data shown in (E) and (F) show that for a given Gō model setup (with either homogeneous or heterogeneous interactions), the *Q*-dependence of energy and entropy exhibits similar trends for Im7 and Im9, indicating that the nature of the Im7 and Im9 equilibrium intermediates (observed in the models with MJ Gō interactions) are rather similar. In both cases, the second barrier at *Q* ≈ 0.9 in the MJ Gō model arises from a decrease in conformational entropy with respect to increasing *Q* (from ≈ 0.8 to ≈ 0.9) that is not fully compensated by a corresponding decrease in energy. (G, H) Snapshots of conformations with *Q* values corresponding to the thermodynamic intermediate states in the models with MJ Gō interactions for Im7 (G) and Im9 (H). The blue and red spheres correspond, respectively, to the N- and C-termini of the conformations. Snapshots for the models with homogeneous Gō and MJ Gō interactions are depicted by green and red traces respectively. The black traces represent the PDB structures of Im7 (G) and Im9 (H). The *Q* value for the Im7 snapshots [green and red traces in (G)] is *Q* = 0.838, that for the Im9 snapshots [green and red traces in (H)] is *Q* = 0.762. These drawings show quite clearly that the intermediate Im7 and Im9 conformations in the MJ Gō models are largely native. The only regions that deviate significantly from the native conformation are a short disordered C-terminal segment for Im7 (G) and short disordered N- and C-terminal segments for Im9 (H). Comparing the red and green traces in (G) and (H) indicates that the equilibrium intermediates in the Im7 and Im9 models with MJ Gō interactions are a consequence of these models’ significantly higher degree of disorder of the C-terminal region relative to that in models with homogeneous Gō interactions. The C-terminal regions are more disordered in the MJ Gō models because each of the amino acid sequences for the regions (GKPGFKQG for Im7 and GKSGFKQG for Im9) contains only one hydrophobic residue. As a result, the favorable interaction between the C-terminal sequence and the rest of the protein is weak when MJ energies are used for the Gō interactions. As discussed in the main text, the present results in this figure may be compared with those reported in Fig 7 of Karanicolas and Brooks [Karanicolas J, Brooks CL (2003) Improved Gō-like models demonstrate the robustness of protein folding mechanisms towards non-native interactions. *J Mol Biol* 334:309–325].
(EPS)

S4 Fig Mutational effects on Im9 folding. Results are for db+MJ*hϕ* [(A) and (C)] and a variant of the model [WT*; (B) and (D)] for Im9 in which the strength of the nine native contact interactions of L33 is reduced by one half. (A, B) Native (lower right) and nonnative (upper left) contact probability maps (color scale on right) for conformations with 0.8 < *Q* < 0.9 along folding trajectories of the V37L/E41V/V71I triple mutant in the two models simulated under strongly folding conditions similar to those in Fig 4 of the main text [*ϵ*/*k*_*B*_ *T* = 1.42 for (A) and 1.45 for (B)]. As in Fig 5 of the main text, the maps provide occurrence probabilities of contacts in a putative intermediate-state ensemble. (C) Kinetic FP profiles (as in Fig 4D in the main text) of the wildtype (WT), the V37L/V71I double mutant, and the V37L/E41V/V71I triple mutant in the db+MJ*hϕ* model. (D) Same as (C) but in the above-defined variant of the db+MJ*hϕ* model. Note that the wildtype (WT*) profile in this model is different from that shown in (C).
(EPS)

S5 Fig Folding behaviors in variants of the db+*hϕ* model with homogeneous nonnative hydrophobic interactions. Kinetic FP profiles (defined as in Fig 4C and 4D of the maintext) under folding conditions corresponding to zero denaturant concentration are shown for variants of the db+*hϕ* model of (A) Im7 and (B) Im9 with uniform *κ*_*ij*_ = −1.0 but different values for the overall hydrophobic strength *K*_HP_ = 1.1 (blue curves), 1.2 (magenta curves), and 1.3 (red curves). Shown in black are the *K*_HP_ = 1 kinetic FP profiles for the original db+*hϕ* model; theses black profiles correspond to the dashed curves in Fig 4C and 4D of the maintext. The native interaction strengths used to compute the present model zero-denaturant results for *K*_HP_ = 1.0, 1.1, 1.2, and 1.3 are, respectively, *ϵ*/*k*_*B*_ *T* = 1.45, 1.47, 1.54, and 1.64 for Im7 (A); and *ϵ*/*k*_*B*_ *T* = 1.36, 1.38, 1.40, and 1.45 for Im9 (B).
(EPS)

S6 Fig Conformational distributions under different restraining potentials. The distributions of model Im7 conformational population under restraining (bias) potential $V_{\text{bias}}(Q,Q_{0})=K_{Q}{\tilde{Q}}_{\text{n}}^{2}{\left(Q-Q_{0}\right)}^{2}$ at simulation temperature *T* = 0.704 (free energy of folding Δ*G* ≈ −4.1*k*_*B*_ *T*) with *K*_*Q*_ = 0.025 (A), 0.05 (B), 0.075 (C), and 0.1 (D) are shown for the 64 equally-spaced *Q*_0_ values used in this work. *K*_*Q*_ = 0.1 is used to obtain the results in the main text. Note that all distributions for individual *Q*_0_ are Gaussian-like for *K*_*Q*_ = 0.05, 0.075, and 0.1 (B, C, and D); but some of the distributions at higher values of *Q*_0_ are clearly non-Gaussian for *K*_*Q*_ = 0.025 (A).
(PDF)

S7 Fig Coordinate-dependent diffusion coefficient *D*(*Q*) for the present model Im7 at simulation temperature *T* = 0.704 is estimated using 64 different *Q*_0_ values (as in S3 Fig) for *K*_*Q*_ = 0.05 (green), 0.075 (red), and 0.1 (black). The resulting *D*(*Q*) functions are very similar within this range of *K*_*Q*_ values.
(PDF)

S8 Fig Coordinate-dependent diffusion coefficient for chymotrypsin inhibitor 2 (CI2). (A) Conformational distributions of model CI2 under restraining potentials *V*_bias_(*Q*, *Q*_0_) with *K*_*Q*_ = 0.01 and *Q*_0_ = 0.1, 0.2, … 0.9. (B) The *D*(*Q*) functions for CI2 we computed using the restraining potential method (*K*_*Q*_ = 0.01) at the indicated simulation temperatures *T* = 1.00, 1.05 (approximate folding-unfolding transition midpoint), and 1.08 (circles connected by solid lines) are compared with that reported for the transition midpoint in Fig 3a of Xu et al. [Xu W, Lai Z, Oliveira RJ, Leite VBP, Wang J (2012) Configuration-dependent diffusion dynamics of downhill and two-state protein folding. *J Phys Chem B* 116:5152–5159] (squares connected by dotted lines). Our *D*(*Q*) (circles) is given in units of reciprocal number of simulation time steps (left vertical scale) whereas the unit for the *D*(*Q*) from Xu et al. (squares, right vertical scale) follows theirs. To facilitate comparison, our results were obtained using the same Gō-like (no-db) model as that given in Eq (1) of Xu et al. Each of our *D*(*Q*) values for a restraining potential centered at *Q*_0_ is plotted at the *a posteriori* average *Q*-position (which is slightly different from *Q*_0_) among the constrained conformations. Our *D*(*Q*) at transition midpoint (*T* = 1.05) matches well with that reported by Xu et al.
(PDF)
