# Supplementary figures and images for: Native Contact Density and Nonnative Hydrophobic Effects in the Folding of Bacterial Immunity Proteins
Source: PLoS Comput Biol. 2015 May 27;11(5):e1004260. doi: 10.1371/journal.pcbi.1004260 (PMC4446218; doi:10.1371/journal.pcbi.1004260)

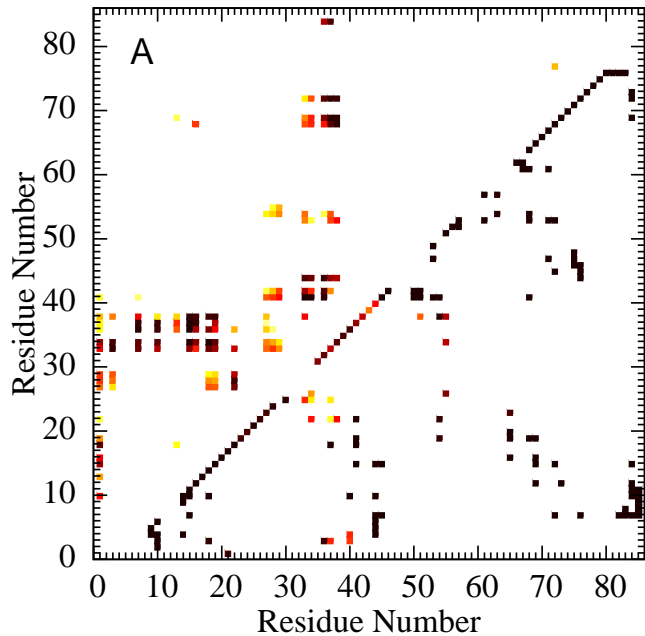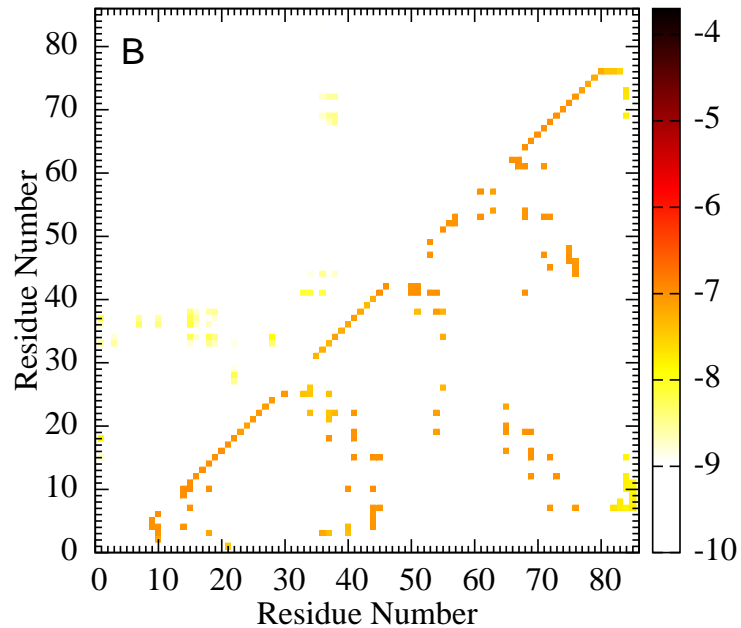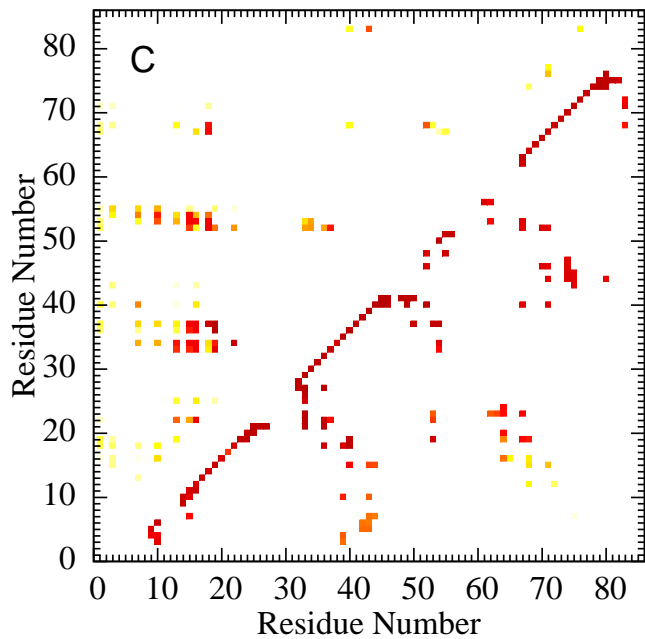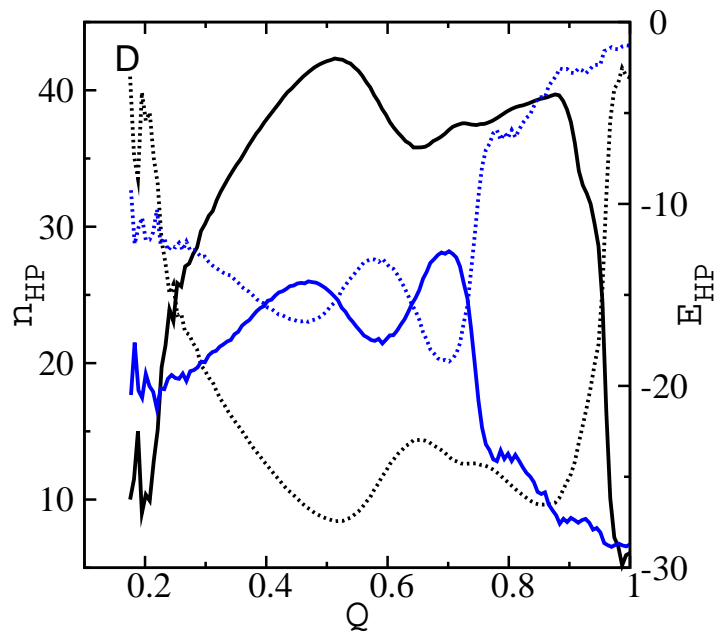

Supplement: S1 Fig — Results here are derived from kinetic folding trajectories simulated at ΔG/k B T values corresponding to the zero-denaturant stabilities of the proteins being modeled. (A–C) Natural logarithm of contact probability (ln P ij, which is normalized for all conformations along folding trajectories, note that this normalization is different from that in Fig 5A). Native and nonnative contacts are shown, respectively, in the lower-right and upper-left (below and above the main diagonal). (A, B) Contact probability maps of Im7 conformations with 0.8 < Q < 0.9 simulated using the db+MJhϕ (A) and db+hϕ (B) models. (C) Contact probability map of Im9 conformations with 0.6 < Q < 0.8 in the db+MJhϕ model. It is clear from these maps that among conformations with Q ≈ 0.8, there are more nonnative contacts in the db+MJhϕ model for Im7 than either the db+hϕ model for Im7 or the db+MJhϕ model for Im9. (D) Number of nonnative hydrophobic contacts (solid curves, left vertical scale) and total nonnative hydrophobic interaction energy E HP (dotted curve, right vertical scale) in the db+MJhϕ model for Im7 (black curves) and Im9 (blue curves) as functions of Q. (PDF) [file pcbi.1004260.s001.pdf]

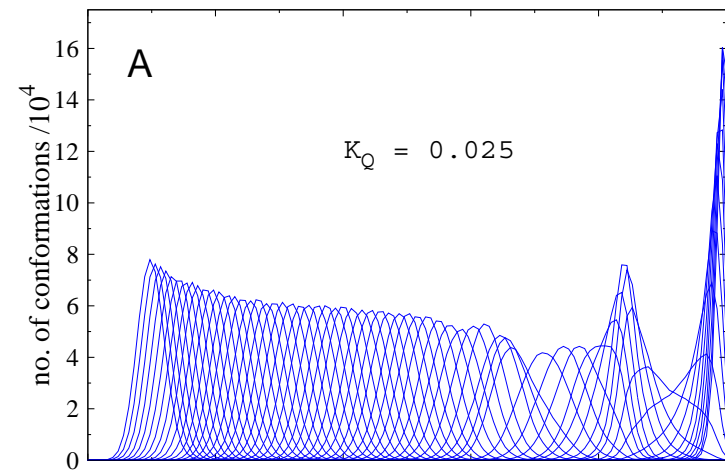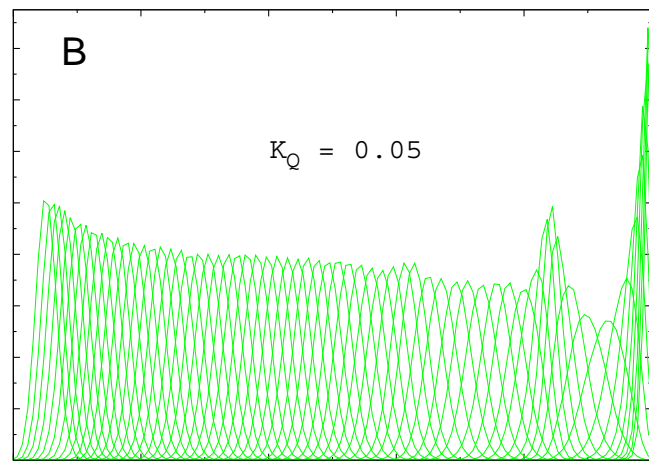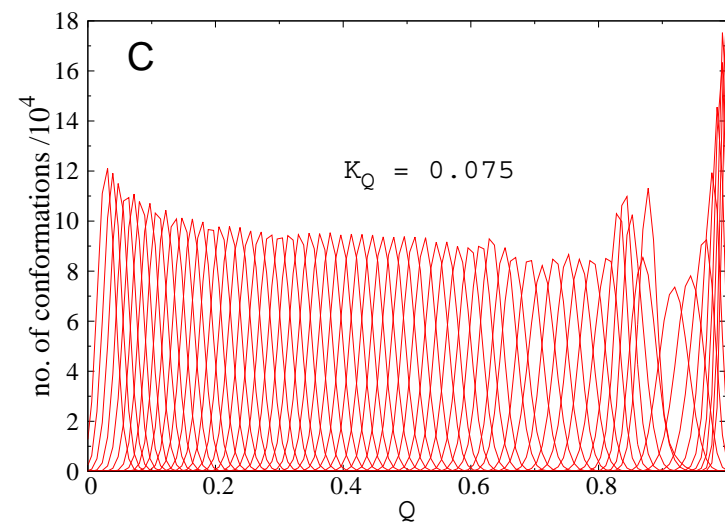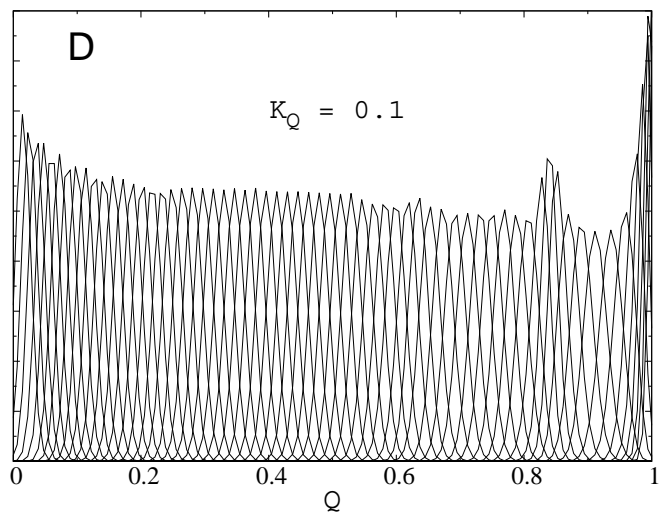

Supplement: S6 Fig — The distributions of model Im7 conformational population under restraining (bias) potential Vbias(Q,Q0)=KQQ˜n2(Q−Q0)2 at simulation temperature T = 0.704 (free energy of folding ΔG ≈ −4.1k B T) with K Q = 0.025 (A), 0.05 (B), 0.075 (C), and 0.1 (D) are shown for the 64 equally-spaced Q 0 values used in this work. K Q = 0.1 is used to obtain the results in the main text. Note that all distributions for individual Q 0 are Gaussian-like for K Q = 0.05, 0.075, and 0.1 (B, C, and D); but some of the distributions at higher values of Q 0 are clearly non-Gaussian for K Q = 0.025 (A). (PDF) [file pcbi.1004260.s006.pdf]

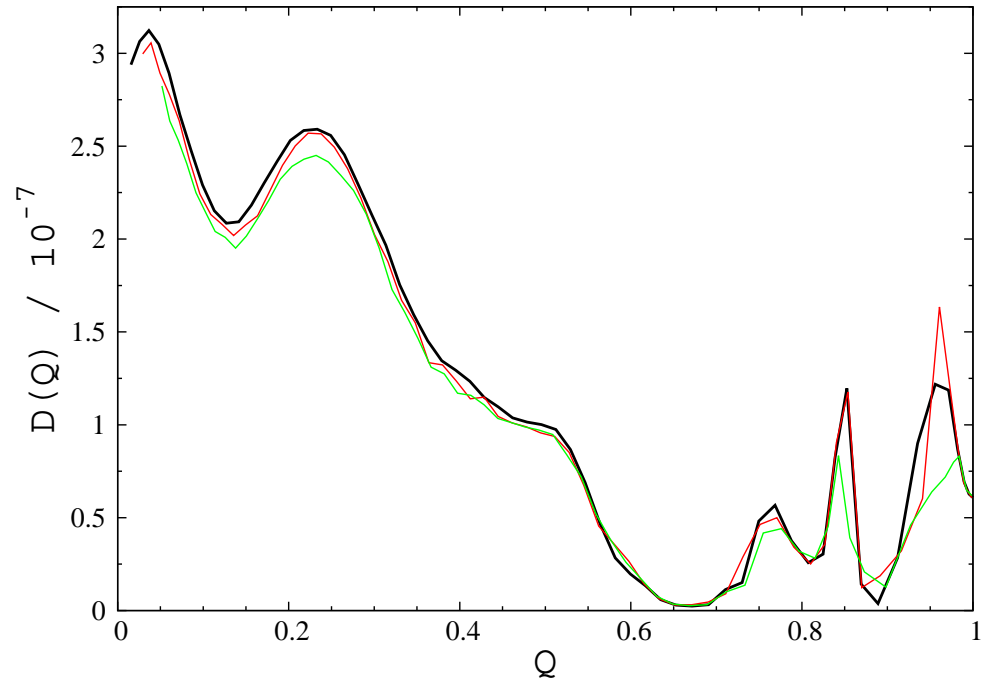

Supplement: S7 Fig — The resulting D(Q) functions are very similar within this range of K Q values. (PDF) [file pcbi.1004260.s007.pdf]

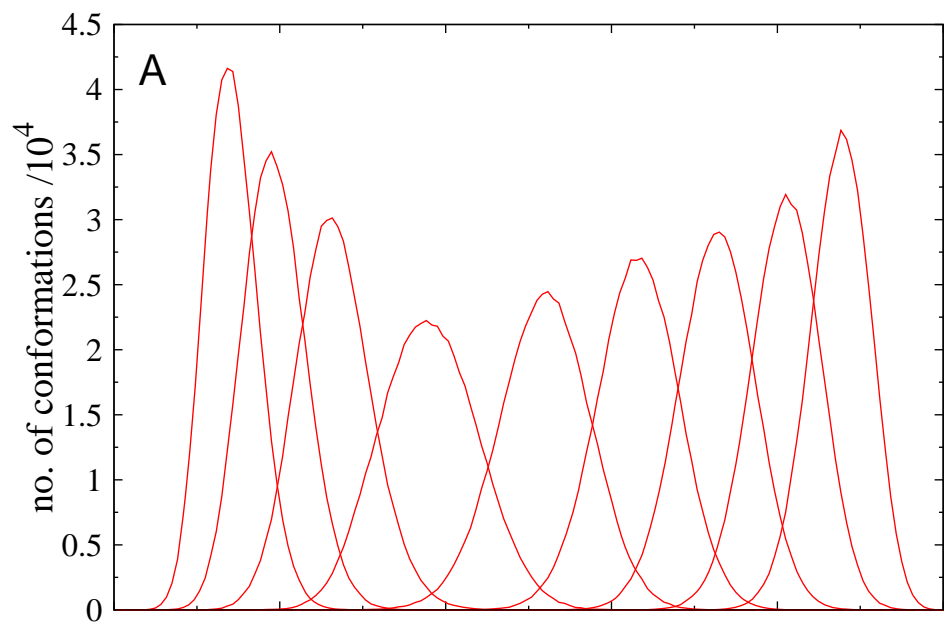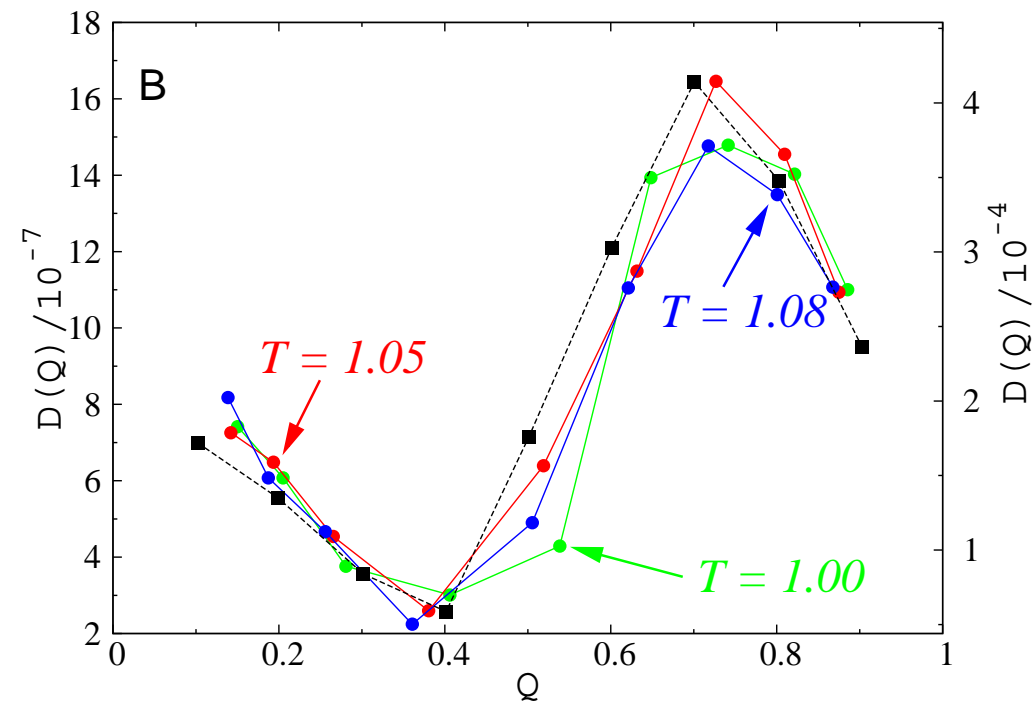

Supplement: S8 Fig — (A) Conformational distributions of model CI2 under restraining potentials V bias(Q, Q 0) with K Q = 0.01 and Q 0 = 0.1, 0.2, … 0.9. (B) The D(Q) functions for CI2 we computed using the restraining potential method (K Q = 0.01) at the indicated simulation temperatures T = 1.00, 1.05 (approximate folding-unfolding transition midpoint), and 1.08 (circles connected by solid lines) are compared with that reported for the transition midpoint in Fig 3a of Xu et al. [Xu W, Lai Z, Oliveira RJ, Leite VBP, Wang J (2012) Configuration-dependent diffusion dynamics of downhill and two-state protein folding. J Phys Chem B 116:5152–5159] (squares connected by dotted lines). Our D(Q) (circles) is given in units of reciprocal number of simulation time steps (left vertical scale) whereas the unit for the D(Q) from Xu et al. (squares, right vertical scale) follows theirs. To facilitate comparison, our results were obtained using the same Gō-like (no-db) model as that given in Eq (1) of Xu et al. Each of our D(Q) values for a restraining potential centered at Q 0 is plotted at the a posteriori average Q-position (which is slightly different from Q 0) among the constrained conformations. Our D(Q) at transition midpoint (T = 1.05) matches well with that reported by Xu et al. (PDF) [file pcbi.1004260.s008.pdf]
